# From *Aloe vera* Leaf Waste to the Extracts with Biological Potential: Optimization of the Extractions, Physicochemical Characterization, and Biological Activities

**DOI:** 10.3390/plants12142744

**Published:** 2023-07-24

**Authors:** Muna Rajab Elferjane, Aleksandra A. Jovanović, Violeta Milutinović, Natalija Čutović, Milica Jovanović Krivokuća, Aleksandar Marinković

**Affiliations:** 1 Faculty of Nursing and Health Sciences, University of Misurata, Alshowahda Park, 3rd Ring Road, Misurata 2478, Libya; muna_rajab2014@yahoo.com; 2 Faculty of Technology and Metallurgy, University of Belgrade, Karnegijeva 4, 11000 Belgrade, Serbia; marinko@tmf.bg.ac.rs; 3 Institute for the Application of the Nuclear Energy INEP, University of Belgrade, Banatska 31b, 11080 Belgrade, Serbia; milicaj@inep.co.rs; 4 Faculty of Pharmacy, University of Belgrade, Vojvode Stepe 450, 11000 Belgrade, Serbia; violeta.milutinovic@pharmacy.bg.ac.rs; 5 Institute for Medicinal Plant Research “Dr Josif Pančić”, Tadeuša Košćuška 1, 11000 Belgrade, Serbia; ncutovic@mocbilja.rs

**Keywords:** *Aloe vera*, biological potential, extraction, optimization, polyphenols

## Abstract

In the study, the optimization of the extraction from *Aloe vera* leaf waste was performed via varying solid-to-solvent ratio, solvent type, extraction time, and technique (maceration, heat-, ultrasound-, and microwave-assisted extractions—HAE, UAE, and MAE, respectively). The optimal extraction conditions for achieving the highest polyphenol content are a 1:30 ratio, 70% ethanol, and 30 min of HAE. Total flavonoid and protein contents were significantly higher in the extract from MAE, while total condensed tannin content was the highest in HAE. LC-MS analysis quantified 13 anthraquinone and chromone compounds. The variations in the FT-IR spectra of the extracts obtained by different extraction procedures are minor. The influence of extraction conditions on the antioxidant ability of the extracts depended on applied antioxidant assays. The extracts possessed medium inhibition properties against *Staphylococcus aureus* and weak inhibitory activity against *Enterococcus feacalis*. The extracts had stimulative effect on HaCaT cell viability. Regarding the extraction yield, there was a significant difference between the used extraction techniques (MAE > HAE > maceration and UAE). The presented study is an initial step in the production of polyphenol-rich extracts from *A. vera* leaf waste aimed to be used for the potential preparation of pharmaceutical and cosmetic formulations for the skin.

## 1. Introduction

*Aloe vera* (L.) Burm. f. (Asphodelaceae) is a tropical, drought-resistant, and perennial succulent native to Africa that later spread to other parts of the world. The plant contains anthraquinone glycosides, mostly C-glucosides, such as barbaloin (10–57%) and aloin (4.5–25%), aloesin and its aglycone aloesone, anthraquinone aglycones, resins, mono- and polysaccharides, polypeptides, flavonoids, terpenoids, tannins, sterols, chromones, lectins, fatty, amino and organic acids, enzymes, saponins, vitamins, and minerals [1,2]. Aloe has shown antitumor, antimicrobial, antiviral, anti-inflammatory, antioxidant, hypocholesterolemic, hypoglycemic, antiulcerogenic, immunomodulatory, analgesic, dermal protection, wound healing, burn healing, and frostbite healing activities [1,2,3,4,5,6,7]. Aloe is widely used as an active ingredient in laxative and antiobesity preparations and as a moisturizer, emollient, or wound-healing agent in pharmaceuticals, sunscreen, and other cosmetic formulations [1,8,9]. It has cathartic properties through action on the colon and indirectly stimulates peristalsis via induction of electrolytes and water secretion in the intestinal lumen, inhibition of their reabsorption from the large intestine, and consequently the increase in intestinal volume and filling pressure [1,8]. Carboxypeptidase, a serine carboxypeptidase enzyme, is a primary antithermic agent that showed analgesic activity and dermoprotectant activity against burns [1,4].

Plant waste or by-products possess plenty of active and non-active components that may be applied in various food, functional food, pharmaceutical, or cosmetic formulations. There are different and widely used ingredients isolated from waste, including flavors from apple/carrot pomace, sugar beet pulp, olive, apple, banana, and mango waste, enzymes from the banana peel, lemon skin, cabbage waste, apple, and strawberry pomace, organic acids from vegetable and fruit peel, dietary fibers from onion layers, orange/peach peel, carrot/tomato pomace, apple, and mango waste, polyphenols from chokeberry leaves, grape pomace, pear, apple, peach, and pomegranate peel, etc., [10,11,12]. *A. vera* waste is also rich in carbohydrates, non-essential and essential amino acids, lipids, organic acids, chromones, polyphenols, such as flavonoids and anthraquinones, minerals, vitamins, pigments, and volatile organic compounds [13]. Additionally, according to Globe Newswire [14] and Semerel et al. [13], the mentioned waste is considered high-value due to the potential for extraction of its biologically active components and their various applications, which are estimated to be worth $2.2 billion by 2025 on the global market. Furthermore, Giannakoudakis et al. [15] have reported about *A. vera* waste biomass-based sorbents and modified counterparts in water technology and wastewater treatment process for heavy metal, dye, and other pollutants removal from water.

Biologically active components can be extracted from herbal material quickly and effectively using novel extraction procedures, including ultrasound-assisted extraction [16,17], microwave-assisted extraction [18,19], enzyme-assisted extraction [12,20], accelerated solvent extraction [21], sub- and supercritical fluid extractions [12,22], pressurized liquid extraction [23,24], etc. The mentioned modern techniques provide various benefits, including reducing solvent consumption and extraction time, a higher extraction yield, and high extract quality. Minimizing the negative environmental influence of the consumption of a higher amount of extraction solvents, the novel extraction technologies support the concept of a “green” solvent [17].

The present study aimed to optimize extraction from *A. vera* leaf waste by investigating factors of interest: solid-to-solvent ratio, solvent type, and extraction time. Moreover, four extraction techniques—maceration (as a traditional procedure), heat-assisted extraction (HAE), ultrasound-assisted extraction (UAE) using an ultrasound probe, and microwave-assisted extraction (MAE) using a microwave reactor (as modern procedures)—were employed. In the study, four different extraction techniques were used with the aim to determine the optimal type of extraction, i.e., to perform a selection between traditional methods (which are simple and non-expensive, but prolonged) and modern methods (which require expensive devices but provide faster kinetics), with the application of optimal extraction conditions. Optimization of the extraction processes was performed via polyphenol yield, while qualitative and quantitative analyses of individual compounds were performed using the LC-MS method. Fourier-transform infrared (FT-IR) spectroscopy analysis and determination of total flavonoid, condensed tannin, and protein contents, physical characteristics (extraction yield, zeta potential, conductivity, density, surface tension, and viscosity), antioxidant and antimicrobial properties, as well as effect on viability of keratinocytes were also performed. Hence, the presented study provides evidence of the physicochemical characteristics and biological potential of the extracts from *A. vera* leaf waste that can add value, improve quality, or represent an active ingredient of pharmaceutical and cosmetic formulations for skin application.

## 2. Results

The first step of the present research was the optimization of the extraction from *A. vera* leaf waste through varying different extraction conditions (solid-to-solvent ratio, solvent type, and extraction time) and employing four extraction techniques (maceration, HAE, UAE, and MAE) via analyzing a variable of interest—polyphenol content of the extracts. In the mentioned step, a one-way analysis of variance followed by Duncan’s post hoc test was used to investigate all factors’ influence on total polyphenol content (TPC), while the full factorial design was employed to obtain the optimal conditions (specific to each extraction method) for achieving the highest polyphenol content. The second step was the determination of the antioxidant potential of all prepared extracts via investigation of factor and extraction procedure influences. The third step was the investigation of antimicrobial activity and effect on keratinocyte viability of the selected *A. vera* extracts (the samples obtained under the optimal extraction conditions in every employed extraction technique for achieving the highest polyphenol content). The final step was the determination of physicochemical properties of the selected extracts.

### 2.1. Factor Effects on Total Polyphenols

One-way ANOVA analysis followed by Duncan’s post hoc test were implemented to determine how significant the impact of the extraction conditions (solid-to-solvent ratio, solvent type, and extraction time) was on polyphenol concentration and which factor levels provide the highest TPC. STATISTICA 7.0 software was employed for statistical analysis by one-way ANOVA, where factor effect on polyphenol yield was observed through absolute values of standardized estimated effects. Subsequently, the two most promising levels of each observed factor were selected for further experimental design. In addition, the mentioned statistical tools (one-way ANOVA and Duncan’s test) were used to examine the influence of high temperature, ultrasound waves, and microwaves on polyphenol yield.

#### 2.1.1. Effect of Solid-to-Solvent Ratio on Polyphenol Content

The solid-to-solvent ratio was found to be one of the factors influencing the polyphenol content in the extracts [25,26,27]. The impact of different solid-to-solvent ratios (1:10, 1:20, and 1:30 g/mL) on the TPC is shown in Table 1.

The highest polyphenol concentration in *A. vera* extracts prepared using maceration, HAE, and MAE was achieved during extraction at a 1:30 ratio (9.95 ± 0.51, 9.76 ± 0.27, and 8.39 ± 0.65 mg gallic acid equivalent (GAE)/g, respectively), while in UAE, there was no statistically significant difference between 1:20 and 1:30 ratios (6.29 ± 0.47 and 6.74 ± 0.14 mg GAE/g, respectively). In all extracts, a statistically significant improvement in polyphenol content was noticed as the solid-to-solvent ratio increased. Additionally, in the case of a large amount of herbal matrix (1:10 ratio), a significant drop in TPC in all used extraction techniques can be noticed (5.23 ± 0.70 mg GAE/g for maceration, 6.61 ± 0.47 mg GAE/g for HAE, and 6.15 ± 0.74 mg GAE/g for MAE) and particularly in UAE (3.51 ± 0.19 mg GAE/g).

Since TPC has achieved maximal yield with a 1:30 ratio, followed by a 1:20 ratio in all employed extraction techniques, these factor levels were selected for the further factorial design.

#### 2.1.2. Effect of Solvent Type on Polyphenol Content

The results of the effect of three solvent types (50 and 70% ethanol, and water) on the polyphenol yield are also shown in Table 1. The results indicate that all tested levels of solvent type in all examined extraction techniques differ significantly. For each extraction method, TPC was significantly lower when water was used as an extraction medium (6.07 ± 0.18, 7.86 ± 0.08, 5.22 ± 0.17, and 7.61 ± 0.62 mg GAE/g). Furthermore, the highest polyphenol concentration was achieved using 50% ethanol in maceration (9.47 ± 0.78 mg GAE/g), while in HAE and UAE, the highest TPC was detected in 70% ethanol extracts (10.40 ± 0.28 and 9.24 ± 0.34 mg GAE/g, respectively). Nevertheless, in MAE, there was no statistically significant difference between 50 and 70% ethanol extracts and TPC amounted to ~9 mg GAE/g.

Since the criteria for selecting the factor levels for further analysis was a maximum of TPC, 50% and 70% ethanol were selected to be included in the full factorial design.

#### 2.1.3. Effect of Extraction Time on Polyphenol Content

The influence of three different extraction times (depending on the extraction technique) on polyphenol concentration is presented in Table 1 as well. Namely, the impact of time was conducted in maceration at 30, 45, and 60 min, in HAE at 15, 30, and 45 min, in UAE at 5, 15, and 30 min, and in MAE at 1, 2, and 3 min. The extraction time had a significant impact on the polyphenol content of the extracts (except in the case of HAE). In our preliminary screening, a slight decline in polyphenol yield was noticed at 90 and 120 min of maceration (data not shown). Additionally, after 60 and 90 min of HAE, a significant drop of TPC in *A. vera* extracts was noticed (data not shown). The obtained results are in agreement with the literature data, where prolonged extraction time caused a decrease in polyphenol concentration in ethanol extracts at 60 and 90 min [28,29]. As can be seen from Table 1, the extraction time had a significant impact on the TPC of *A. vera* extracts (except in the case of HAE). The maximum TPC was achieved at 45 min in maceration (9.66 ± 0.51 mg GAE/g), whereas polyphenol yield was significantly lower after 30 and 60 min (8.31 ± 0.72 and 8.43 ± 0.38 mg GAE/g, respectively). In HAE, there were no significant changes in the TPC of the extracts prepared at different extraction times, and polyphenol concentration varied in a very narrow range (9.57–9.95 mg GAE/g). The extracts obtained after 5 min of UAE were a poor source of polyphenols (7.44 ± 0.12 mg GAE/g), while there was no statistically significant difference between 15 and 30 min (8.03 ± 0.23 and 8.07 ± 0.24 mg GAE/g, respectively). The MAE extracts prepared at 1 min possessed significantly lower TPC (7.64 ± 0.40 mg GAE/g) and the extracts obtained after 2 and 3 min of microwave irradiation showed statistically significantly higher polyphenol yield (8.70 ± 0.42 and 8.82 ± 0.64 mg GAE/g, respectively).

Since the highest TPC in maceration was obtained at 45 min and there was no significant difference between 30 and 60 min; to minimize energy cost, 30 and 45 min were subjected for further statistical analysis. Following the same logic, 15 and 30 min of HAE (there was no significant difference between all used extraction times), 5 and 15 min of UAE (there was no significant difference between 15 and 30 min), and 1 and 2 min of MAE (there was no significant difference between 2 and 3 min) were chosen for further statistical analysis.

#### 2.1.4. Effect of High Temperature on Polyphenol Content

In our preliminary screening, the influence of increased temperature (40–80 °C) was examined and polyphenol content became higher with increasing temperature (data not shown). According to these findings, 80 °C was maintained for the further experiment of optimization of HAE. The obtained results are following the Miron et al. findings [30].

The TPC in *A. vera* extracts obtained at high temperature was significantly higher than in the case of the extracts prepared at room temperature, for almost all observed factor levels (except in the case of a 1:30 ratio and 50% ethanol, where there were no statistically significant differences) and in the case of extracts prepared using UAE and MAE at all examined factor levels (Table 1).

**Figure 1:**
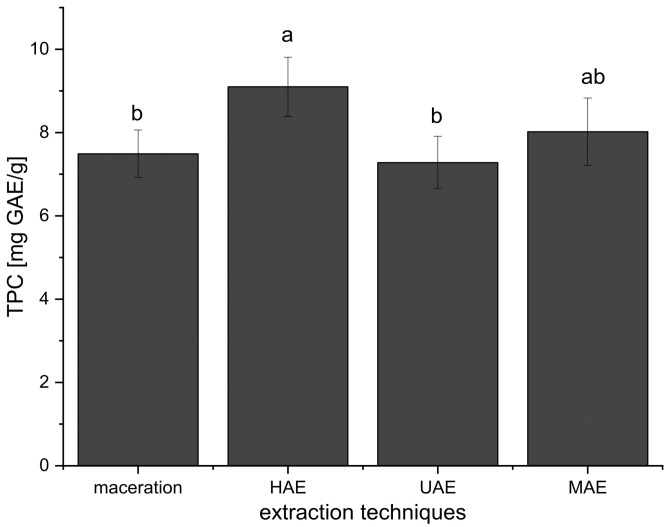
Effects of extraction techniques on total polyphenol content (TPC) of all prepared *Aloe vera* extracts using maceration, heat-, ultrasound-, and microwave-assisted extractions (HAE, UAE, and MAE, respectively); different letters represent different population groups based on Duncan’s post hoc test (*p* < 0.05).

Furthermore, the mean TPC value of all extracts prepared using HAE (all TPCs added together and expressed as mean ± standard deviation) was significantly higher in comparison to TPCs of all extracts obtained in maceration and UAE, while there was no significant difference compared to the extracts from MAE (Figure 1). Even at the first 15 min of HAE, the TPC was higher (15–19%) than TPC in the extracts prepared using maceration (30 min) and UAE (15 and 30 min) (Table 1).

#### 2.1.5. Effect of Ultrasound Waves on Polyphenol Content

The influence of ultrasound waves on TPC at all examined factor levels is presented in Table 1 as well. The comparison of the mean TPC value of all *A. vera* extracts prepared in UAE and three other employed extraction procedures is presented in Figure 1. In the preliminary screening (data not shown), the amplitude was evaluated at three levels (20, 40, and 60%). The highest polyphenol yield was obtained at 60% amplitude; thus, this level was maintained for further experiments of UAE.

The results have shown that TPC was higher only in the extracts prepared using 70% ethanol in UAE compared to their parallels prepared in maceration (Table 1). The content of polyphenols was the same in the extracts after 15 min of UAE and after 30 min of maceration. Namely, as can be seen from Figure 1, the TPC values of the extracts obtained using UAE were not significantly different in comparison to maceration and MAE but were significantly lower than in the case of HAE.

#### 2.1.6. Effect of Microwaves on Polyphenol Content

In the preliminary screening (data not shown), different temperatures in a microwave reactor (60–160 °C) were examined for the extraction from *A. vera* waste. The highest polyphenol content was obtained in a range of 100–160 °C, thus 100 °C was maintained for the future experiment of MAE, which is in accordance with literature data [31].

Comparing the extracts prepared using maceration and MAE, it can be concluded that MAE gave a higher TPC only in 70% ethanol and water extracts, while between other examined factor levels there was no statistically significant difference (Table 1). The TPC values in the extracts obtained in HAE and MAE were similar for 1:10 ratio and 50% ethanol and water, while at 1:20 and 1:30 ratios and in 70% ethanol extracts, HAE gave a significantly higher polyphenol content (Table 1). The results also showed that TPC was higher in the extracts obtained using MAE compared to UAE at all observed factor levels (except in the case of 70% ethanol extracts where the TPC values were not significantly different, Table 1). According to the results from Figure 1, the mean TPC value of all extracts prepared using MAE was not significantly different in comparison to maceration, HAE, and UAE.

### 2.2. Experimental Design

**Figure 2:**
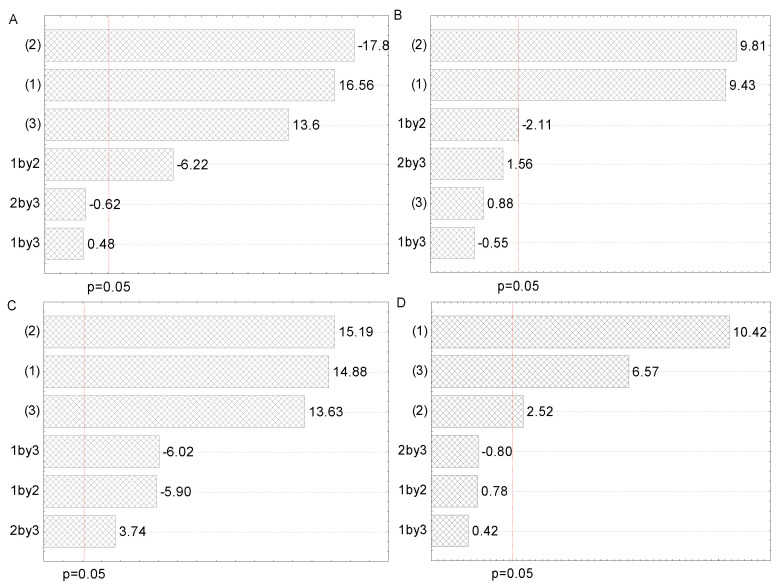
Pareto charts of the influence of factors and their interactions on the polyphenol concentration in (**A**) maceration, (**B**) heat-, (**C**) ultrasound-, and (**D**) microwave-assisted extractions from *Aloe vera* leaf waste: solid-to-solvent ratio (1), solvent type (2), and extraction time (3).

In the present study, experimental design (2^3^ full factorial design) was employed to determine the best combination of extraction variables (solid-to-solvent ratio, solvent type, and extraction time). Following the results of the screening study, solid-to-solvent ratio (1:20 and 1:30 g/mL), solvent type (50 and 70% ethanol), and extraction time (30 and 45 min for maceration, and 15 and 30 min for HAE, 5 and 15 min for UAE, and 1 and 2 min for MAE), each factor at two levels, were used for the experimental design of the extraction optimization. The influence of the two most promising levels of each of the three observed factors selected for 2^3^ factorial design, as well as their interactions, are presented on Pareto charts with the level of significance set at *p* < 0.05 (Figure 2). The effects and corresponding regression coefficients of factors and factor interactions are presented in Supplementary Table S1. The observed and predicted means for the dependent variable (TPC) are listed in Table 2.

All three examined factors have been represented through two selected levels in 2^3^ full factorial design. The Pareto charts in Figure 2 represent all factors that influence the polyphenol content in all four employed extraction procedures. The purpose of this graphical presentation was to show which factors and their interactions had a significant impact on polyphenol content. The lengths of the horizontal bars are proportional to the absolute magnitude of the estimated effects, while the dashed line represents the minimum value of statistically significant effects concerning the dependent variable, TPC (95% of the confidence interval).

As can be seen from Figure 2A, solvent type (factor 2) and solid-to-solvent ratio (factor 1) were the most significant factors for the polyphenol recovery in maceration, whereas extraction time (factor 3) and interaction between solid-to-solvent ratio and extraction time had a lower, but also important, impact. However, the influence of interactions between the solid-to-solvent ratio and the solvent type and between solvent type and extraction time were not significant. According to the literature, solvent type and solid-to-solvent ratio were significant factors for obtaining maceration extracts with a high polyphenol yield from different plant sources [25,27]. From Figure 2B, it can be concluded that solvent type and solid-to-solvent ratio had a significant impact on polyphenol recovery in HAE, followed by the interaction between solid-to-solvent ratio and solvent type, while extraction time and other factor interactions did not show a significant effect on polyphenol concentration. The obtained results are in agreement with the literature, where solvent type and solid-to-solvent ratio significantly influenced polyphenol yield in henna and parsley extracts prepared using HAE [29,32]. It can be observed from Figure 2C that the impact of examined factors in UAE was the same as in the case of maceration, while the influence of their interactions was different. Namely, all interactions significantly influenced the polyphenol content in UAE. Similar to our findings, the solid-to-solvent ratio was the first, while ethanol concentration was the second relevant factor that affected the UAE of polyphenols from desert plants [26]. It can be noticed from Figure 2D that the solid-to-solvent ratio, followed by extraction time and solvent type, possessed a significant influence on the TPC of the extracts prepared using MAE, while all interactions did not have a significant effect. According to the literature, solid-to-solvent ratio, ethanol percentage in the extraction medium, and extraction time had a significant effect on TPC in wild thyme extracts obtained in MAE [19].

Full factorial design (2^3^) was applied to examine the effect of factors on the TPC and to select the optimal conditions for achieving the highest polyphenol yield for each extraction technique separately. In addition, the mentioned statistical method provided the evaluation of the interactions between the investigated factors. The effects and corresponding regression coefficients of factors and interactions of two factors and probability for 2^3^ full factorial design are presented in Supplementary Table S1, while the observed and predicted values of polyphenol content, as the results of the 2^3^ full factorial design, i.e., the best combination of extraction factors’ level for each employed extraction technique, are presented in Table 2.

In the 2^3^ factorial design (three independent variables which had significant effects on TPC, solid-to-solvent ratio, solvent type, and extraction time, each at two levels), the investigation of the factor impact on TPC, as well as the selection of the optimal conditions for achieving the highest polyphenol concentration for all used extraction techniques, were performed (Table 2 and Table 3). Furthermore, the 2^3^ factorial design has also allowed the examination of the interactions between the independent variables.

As can be seen from the table in Supplementary Table S1, the solvent type was the most important factor for TPC of *A. vera* extracts from maceration, but with a negative value. It means that a lower level (50% ethanol in this case) gave better results (higher TPC). Further, the solid-to-solid ratio was the second relevant factor for achieving the highest TPC in maceration and had a positive value, which means that the upper level (1:30 ratio in this case) provided higher polyphenol yield. The extraction time and interaction between solid-to-solvent ratio and extraction time were the third and fourth relevant factors for TPC in maceration, whereas the influence of the other two interactions was not decisive. In HAE, solvent type and solid-to-solvent ratio were the dominant factors, as well as the interaction between them (Supplementary Table S1), which indicated that the impact of solid-to-solvent ratio was not the same at all solvent types. When the interaction between factors is significant, it means that the influence of one factor depends on the used level of the other factor. In the present case, the effect of the solid-to-solvent ratio depends on used solvent type levels. On the other hand, the influence of the extraction time and the interactions between the solid-to-solvent ratio and extraction time and the solvent type and extraction time was not significant. In UAE, all observed factors were relevant, followed by the significant interaction between solid-to-solvent ratio and solvent type, whereas the other two interactions were not significant (Supplementary Table S1). Additionally, all examined factors had a positive effect on the TPC (a higher level of factor provides better results) of the extracts obtained in MAE, while their interactions were not relevant (Supplementary Table S1).

The results of the 2^3^ full factorial design presented as the observed and predicted values of polyphenol concentration and the combination of the extraction factors’ level for achieving the highest polyphenol content are shown in Table 2. In maceration, the highest TPC (observed value) was reached using the highest solid-to-solvent ratio (1:30) and 50% ethanol after 45 min (10.09 ± 0.18 mg GAE/g). The model has predicted the maximal TPC under the same conditions (10.11 mg GAE/g). The highest polyphenol content (observed value) in HAE was measured in a combination of a 1:30 ratio and 70% ethanol after 30 min (10.42 ± 0.10 mg GAE/g). The model has predicted the maximal TPC under the same operational conditions (10.35 mg GAE/g). The highest polyphenol content (observed value) in UAE was observed in combination with a 1:30 ratio and 70% ethanol after 15 min (9.24 ± 0.24 mg GAE/g). The model has predicted the minimal TP yield under the same operational conditions (9.33 mg GAE/g). The highest polyphenol content (observed value) in MAE was observed in combination with a 1:30 ratio and 70% ethanol after 2 min (9.00 ± 0.66 mg GAE/g). The model has predicted the minimal TPC using the same extraction conditions (9.03 mg GAE/g). Since the differences between observed and predicted values were minimal (<1%), a full factorial design can be suggested as an adequate model for optimization of the polyphenol extraction from *A. vera* leaf waste.

According to the presented results, it can be concluded that the optimal extraction conditions and technique for achieving the highest TPC of *A. vera* leaf waste extract are the solid-to-solvent ratio of 1:30, 70% ethanol as an extraction medium, and 30 min of the extraction at high temperature (80 °C).

### 2.3. Total Flavonoid, Condensed Tannin, and Protein Contents of the Extracts

**Figure 3:**
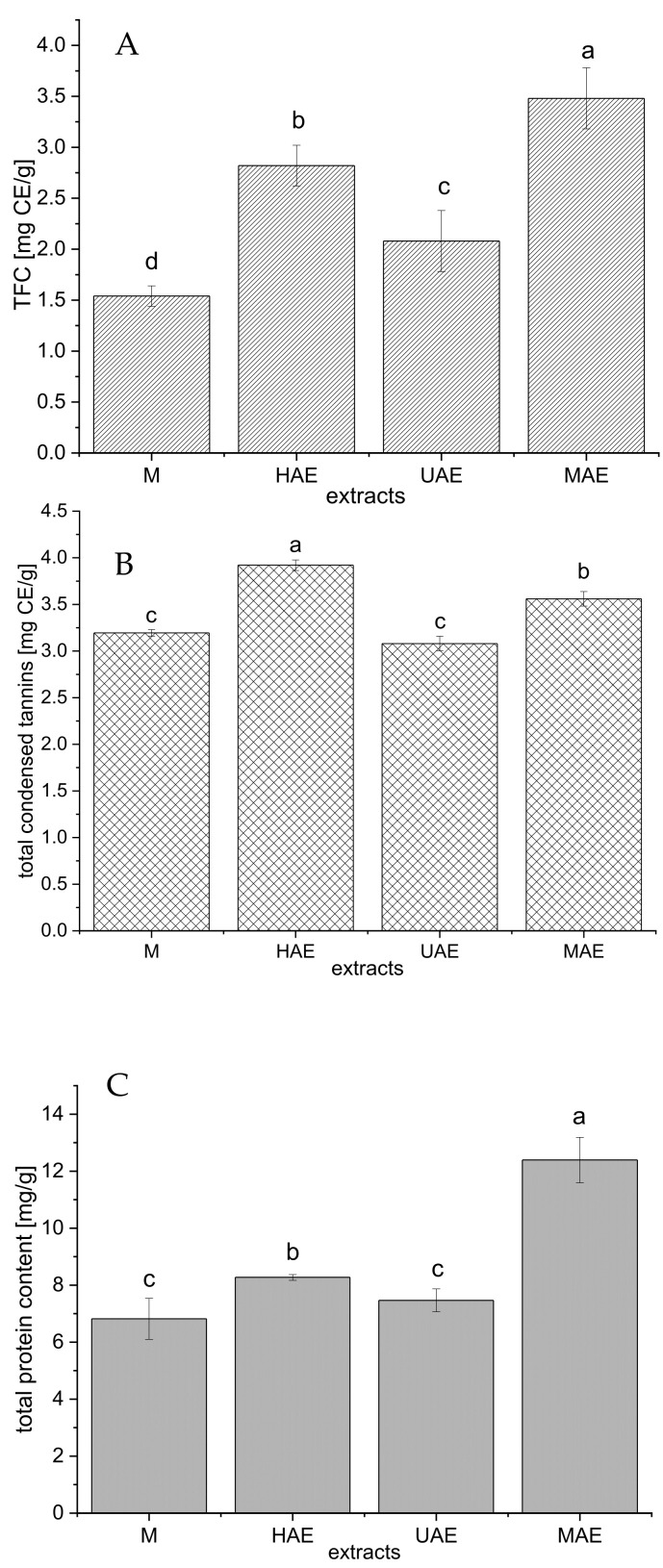
The total flavonoid (**A**), condensed tannin (**B**), and protein (**C**) contents of selected *Aloe vera* waste extracts prepared using maceration, heat-, ultrasound- and microwave-assisted extractions (M, HAE, UAE, and MAE, respectively); TFC, total flavonoid content; CE, catechin equivalent; values with the same letter showed no statistically significant difference (*p* > 0.05; *n* = 3; analysis of variance, Duncan’s post hoc test).

The selected extracts (the samples with the highest polyphenol concentration from all four extraction techniques) were additionally characterized via total flavonoid, condensed tannin, and protein contents; the results are presented in Figure 3.

As can be seen from Figure 3A, the highest total flavonoid content (TFC) was measured in the extract prepared using MAE (3.48 ± 0.3 mg catechin equivalent (CE)/g), followed by HAE (2.82 ± 0.2 mg CE/g) > UAE (2.08 ± 0.3 mg CE/g) > maceration (1.54 ± 0.1 mg CE/g).

The amount of condensed tannin in the extracts is presented in Figure 3B. Namely, the extract prepared using HAE possessed the highest content of condensed tannins (3.92 ± 0.06 mg CE/g), followed by MAE extract (3.56 ± 0.08 mg CE/g), while the extracts obtained in maceration and UAE showed significantly lower concentration of condensed tannins (3.19 ± 0.04 and 3.08 ± 0.08 mg CE/g, respectively).

The content of total proteins follows the trend: MAE > HAE > maceration and UAE (Figure 3C). The extract prepared using a microwave reactor had a statistically significantly higher protein yield (12.4 ± 0.8 mg/g) compared to all other extraction methods. Between the extracts obtained by maceration and ultrasound probe, the differences in the amount of proteins were minimal (6.82 ± 0.72 and 7.47 ± 0.40 mg/g, respectively).

### 2.4. LC-MS Analyses of the Extracts

LC-MS analysis was employed to detect or identify and quantify individual compounds in selected *A. vera* waste extracts and to examine the impact of different extraction techniques on their content. Chromatograms are attached in Supplementary Figure S3a.

#### 2.4.1. Qualitative LC-MS Analysis of the Extracts

Qualitative LC-MS analysis revealed the presence of 13 compounds (Table 3). For this purpose, ESI-MS spectra were recorded in the negative ion mode at a lower of 100 V and a higher voltage of 250 V.

Comparing mass spectral data (Table 3) with the literature [33,34,35], compounds **2**, **4**, **6**–**8**, **10**–**13** were tentatively identified as glycosylated anthraquinones. In mass spectra of some of them (**7**, **13**), a characteristic neutral loss of 162 Da was observed, indicating the presence of a hexose moiety, linked to the aglycone (free anthraquinone) by an *O*-glycosidic bond, e.g., the fragment ion at *m*/*z* 417 [M-162-H]^−^ of **7**. The fragmentation patterns of **2**, **10**, and **11** indicated the structure of C-glycosylated anthraquinones. For the aglycone part of the molecules, the neutral losses of 18 and 42 Da were registered considering the anthraquinones containing a hydroxyl (OH), hydroxymethyl (CH_2_OH) groups, or cleavage of the C—C bonds in tricyclic aromatic structure (9(10H)-anthracenone) in accordance with previously reported fragmentation pathways [33,34,35].

The compounds **10** and **11**, with deprotonated molecules [M-H]^−^ at *m*/*z* 417 and characteristic fragment ions at *m*/*z* 297 and 255 produced by the simultaneous losses of 120 Da and 42 Da, were tentatively characterized as aloin B or A (**10** and **11**). The presence of both diastereoisomers was revealed in analyzed extracts, but in the absence of authentic standards, the order of isomers’ elution can be assumed based on previous investigations [35].

In the mass spectrum of compound **2**, the deprotonated molecule signal was registered at *m*/*z* 447. The fragment ion at *m*/*z* 243 [M-204-H]^-^ indicated the loss of hexose molecule linked to the aglycone by a C-glycosidic bond and simultaneous opening of tricyclic aromatic structure at C-10 (Supplementary Figure S3b), since the C-9 carbonyl group can be stabilized by intramolecular hydrogen bond formed with the C-1 and C-8 phenol groups [34]. The structure of this compound was assigned to hydroxymethyl aloin [35].

The compound **7** was assigned to aloin hexoside due to its *m*/*z* [M-H]^-^ at 579 and fragment ion signal at *m*/*z* 417 presumed by losing hexose moiety (162 Da), where the mass of 417 Da corresponds to aloin structure. Accordingly, compound **6** with [M-H]^−^ at *m*/*z* 813 was considered as an aloin hexoside derivative, regarding fragment ions at *m*/*z* 579 and 417 which originated from aloin hexoside moiety. Another anthraquinone derivative with *m*/*z* [M-H]^−^ at 583 was characterized as an aloe-emodin hexoside derivative (**13**).

By comparing the fragmentation pathways of **5**, **12** with literature data, with the characteristic fragment ion at *m*/*z* 297, it was established that the compounds might be derivatives of anthrone nataloin. The deprotonated molecule of **12** at *m*/*z* 459 indicated the structure of malonyl derivative of nataloin, which was in agreement with previously identified 6′-malonylnataloin A or B in *A*. *vera* leaves methanol extract [35].

Additionally, the fragmentation patterns of 3 and 9 indicated the structure of chromone derivatives malonylaloesin (3) and aloeresin (9) [36,37].

According to the literature data, aloin, as a mixture of aloin A (barbaloin) and aloin B (isobarbaloin), represents an anthraquinone glycoside of the *A. vera* leaves and shows purgative effect [36,37]. The identified compounds in *A. vera* waste extracts are in accordance with the results of the study by Quispe et al. [36].

#### 2.4.2. Quantitative LC-MS Analysis

All detected compounds in *A. vera* extracts were quantified using peak areas in DAD chromatograms recorded at 210 nm. The amounts of these 13 constituents were determined using an emodin calibration curve, for which the regression equation, correlation coefficient (r^2^), concentration range, LOD, and LOQ values are given in Section 4.3.5. The results of the quantitative analysis (expressed as g/100 g of dried extract) are presented in Table 4.

**Figure 4:**
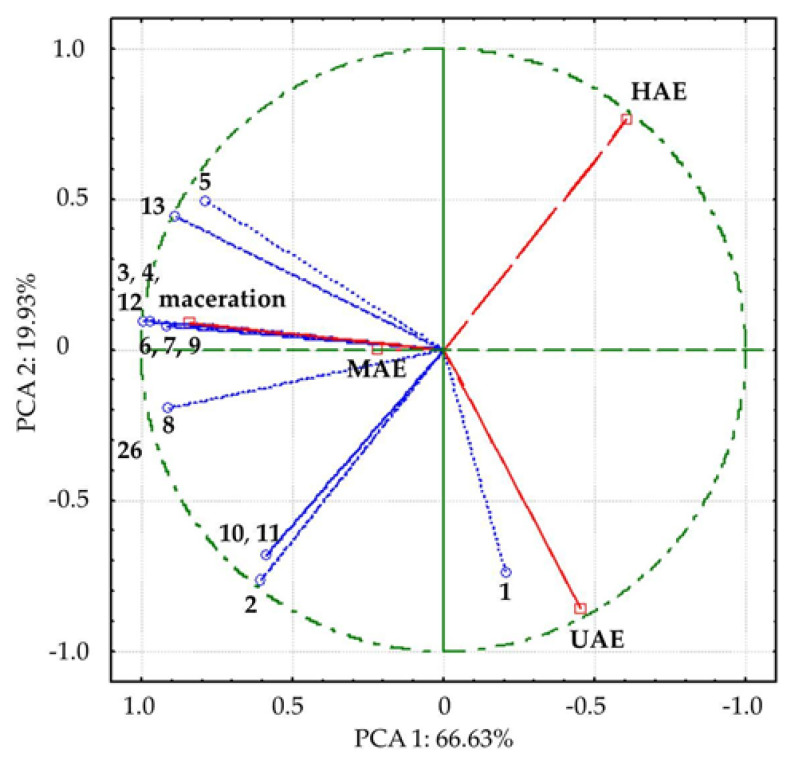
Bi−plot of loading factors of 13 compounds and supplementary scores of analyzed *Aloe vera* leaf waste extracts along the first two principal components. The names of the compounds are presented in Table 3. Empty dots represent detected compounds; empty squares represent the analyzed extracts prepared using maceration, heat-, ultrasound- and microwave-assisted extractions (HAE, UAE, and MAE, respectively).

Among detected compounds, the most abundant anthraquinone was malonylaloesin (3: 0.489–0.188%), followed by hydroxymethyl aloin (2: 0.277–0.114%). Compositions of *A. vera* waste extract prepared using HAE, UAE, and MAE were comparable and different from the macerate with the lowest absolute anthraquinone content. In order to make a clearer distinction between investigated extracts, PCA analysis was performed (Figure 4).

In PCA, all detected compounds correlated with variance, contributing to different positions of investigated extracts, i.e., the extracts obtained in UAE and HAE spatial separation from macerate and MAE extract by first axis (PCA1), along with the separation of UAE extract by second axis (Figure 4). The compounds 3, 4, 8, 12, and 13, which mostly contributed to the first axis, were remarkably positively correlated (0.90 to 1.00) with factor 1, while 1, 2, 10, and 11 were highly negatively correlated (−0.70 to −0.79) with the second axis.

### 2.5. FT-IR Spectra of the Extracts

**Figure 5:**
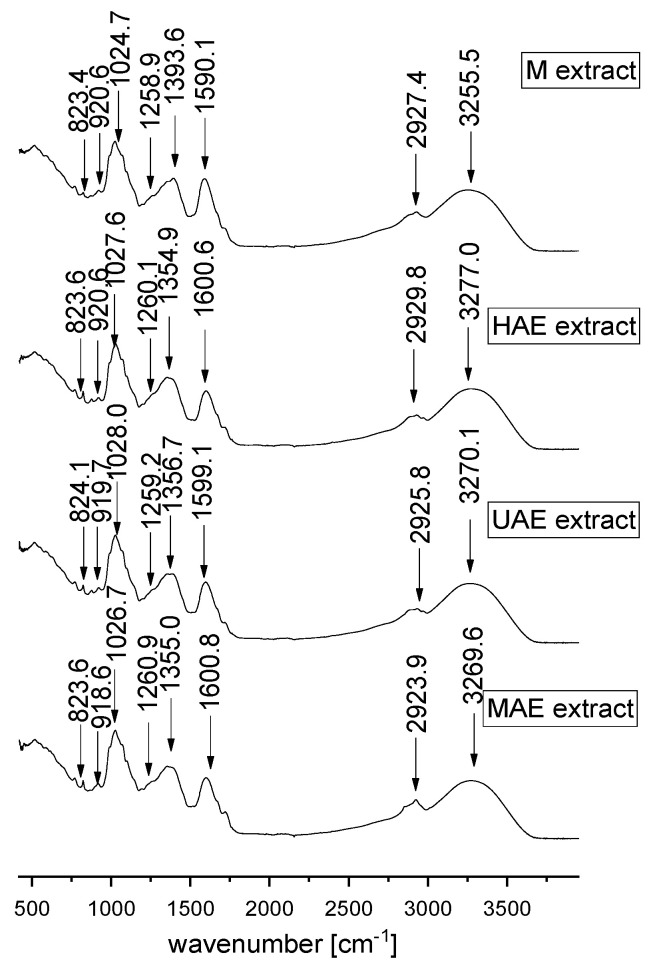
FT-IR spectra of *Aloe vera* leaf waste extracts prepared using maceration, heat-, ultrasound- and microwave-assisted extractions (M, HAE, UAE, and MAE, respectively) in the spectral range from 450 to 4000 cm^−1^.

FT-IR represents a valuable tool for the characterization and identification of compounds or functional groups in an unknown mixture of plant extract. Since FT-IR spectra of pure compounds are unique, as a molecular “fingerprint”, the most common plant components in an unknown mixture can be identified by comparison to a library of known compounds. In the present study, FT-IR analysis was also performed in order to examine the influence of different extraction techniques on the chemical characteristics of selected *A. vera* waste extracts. FT-IR spectra of lyophilized extracts are presented in Figure 5.

In the present study, FT-IR analysis has shown the presence of different biologically active phytochemicals from all examined *A. vera* extracts (the samples with the highest TPC from each extraction procedure). FT-IR spectra of *A. vera* leaf waste extracts (Figure 5) show the mode around the 3000–3500 cm^−1^ range attributed to the stretching vibrations of the OH groups in alcohols and phenols [38,39]. The peak at ~2924–2930 cm^−1^ originates from C-H stretching vibrations of methyl/methylene groups and corresponds to the same vibration identified in wild thyme and green tea extracts [40,41]. The mode at ~1590–1600 cm^−1^ can be related to carboxylic groups and C-C stretch in aromatic rings, which are present in a vast number of plant secondary metabolites and polysaccharide molecules [38,42,43].

Although according to the literature data, the mode at ~1500 cm^−1^ can originate from the extracted flavonoids [44], the absence of the mentioned peak in all examined extracts can be noticed. It also confirms the previously presented results of a quite low flavonoid concentration in the extracts (Section 2.3). In the extract prepared using maceration, the mode at ~1393.6 cm^−1^ can be related to carboxylates [45]; however, the mentioned band was moved to ~1355 cm^−1^ in all other extracts, which can be associated with the presence of OH bending of alcohol and phenol groups and N-O symmetric stretching in nitro compounds [38,42]. The peak at ~1260 cm^−1^ can originate from polyphenol components, i.e., C–C–O vibrations. A group of overlapping modes in the region between 1200 and 800 cm^−1^, peaking at ~1030 cm^−1^, may be attributed to various C–O, C–O–C, and C–C vibrations of alcohols, acids, and sugars. Namely, the peak at ~1025–1027 cm^−1^ is close to the positions of the band originating from C-OH vibrations present in the carbohydrates, the peak at ~920 cm^−1^ can be related to O-H bend of carboxylic acids, whereas the mode of lower intensity at ~824 cm^−1^ can be assigned to monoterpenes, C-H vibrations [42,43,46]. FT-IR spectra showed the presence of various types of functional groups (with their bond types) of biologically active components at different frequencies and rationalize the use of *A. vera* extracts as a herbal remedy. The variations in the FT-IR spectra of the extracts obtained by different extraction procedures are relatively minor.

### 2.6. The Influence of Extraction Factors on Antioxidant Potential

The impact of different solid-to-solvent ratios (1:10, 1:20, and 1:30 g/mL), solvent types (50 and 70% ethanol, and water), and extraction times (depending on the extraction procedures) employing four extraction techniques (maceration, HAE, UAE, and MAE), on antioxidant capacity of the extracts was investigated using four tests (ABTS, DPPH, FRAP, and CUPRAC assays). One-way ANOVA and Duncan’s test were used to examine the impact of solid-to-solvent ratio, solvent type, extraction time, and extraction technique on the antioxidant potential of the extracts. Since all employed antioxidant assays are based on different mechanisms, it is possible to obtain different results, as well as an overall insight into the antioxidant capacity of the extracts. The results of the influence of all examined factors are shown in Supplementary Figure S1 (solid-to-solvent ratio and solvent type), Supplementary Table S2 (extraction time), and Supplementary Figure S2 (extraction procedure).

As can be seen from Supplementary Figure S1A, the solid-to-solvent ratio had a significant impact on ABTS radical scavenging activity in all employed extraction techniques, while solvent type had a significant influence only in HAE and MAE. Namely, ABTS antioxidant activity was higher at a higher solid-to-solvent ratio, as in the case of TPC. Regarding the effect of solvent type on antioxidant potential, in maceration and UAE, all three extraction mediums (50 and 70% ethanol and water) have given extracts with the same antioxidant activity, while in the case of HAE and MAE, the best solvent was 70 and 50% ethanol, respectively. The extraction time did not significantly influence the ABTS antioxidant capacity of the extracts prepared in all employed extraction techniques (Supplementary Table S2). It can be noticed that the obtained results are not in accordance with TPC values at all levels (Section 2.1). In Supplementary Figure S2A, the influence of the employed extraction technique on ABTS antioxidant potential can be seen, expressed as the mean value ± standard deviation of all prepared extracts (MAE ≥ maceration ≥ HAE ≥ UAE).

According to the results from the DPPH assay, it can be concluded that the solid-to-solvent ratio and solvent type had a significant impact on DPPH radical scavenging potential in all employed extraction techniques (Supplementary Figure S1B), while extraction time had a significant influence in UAE and MAE, but only between the lowest level and the other two levels (Supplementary Table S2). As can be seen from Supplementary Figure S1B, ethanol extracts have shown significantly better DPPH radical neutralization ability in comparison to water extracts. In addition, MAE has provided the extracts with a significantly higher radical scavenging potential (lower IC_50_ value) in comparison to other used extraction procedures (Supplementary Figure S2B).

In the FRAP assay, the solid-to-solvent ratio and extraction medium significantly affected the antioxidant potential of the extracts (Supplementary Figure S1C). Namely, the lowest ferric ion-reducing potential was obtained using a 1:10 ratio and water, while the highest activity was shown at a 1:30 ratio in 50 and 70% ethanol extracts. According to the results from the table of Supplementary Table S2, the extraction time had a significant influence on the ferric ion-reducing potential in all employed extraction techniques. However, the influence of time was not the same in all extraction methods. Namely, a longer time of maceration and HAE resulted in a higher potential, but only up to a certain limit (45 min of maceration and 30 min of HAE), while after that time there was no statistically significant difference. In the case of UAE and MAE, the prolonged time provided a higher antioxidant activity (up to 15 min of UAE and 2 min of MAE), but after the mentioned periods, it can be visible a significant drop in antioxidant potential. As can be seen from the figure of Supplementary Figure S2C, the extraction technique significantly affected ferric ion, reducing the capacity of the extracts. FRAP assay is based on the ability of antioxidants, including polyphenol compounds, to reduce Fe^3+^ to Fe^2+^, forming a blue chromophore.

Regarding the results from the CUPRAC assay, in all extraction procedures, solid-to-solvent ratio and solvent type had a significant influence on ion-reducing capacity and it was higher as solvent volume increased and when ethanol/water mixture was used (Supplementary Figure S1D), as in the case of total polyphenols. As can be seen in the table of Supplementary Table S2, extraction time had a significant impact on the ion-reducing capacity of the extracts prepared using maceration, UAE, and MAE. Namely, prolonged time induced higher CUPRAC values in the mentioned extraction procedures, but only up to a certain limit, which was in accordance with the TPC value as well. Additionally, MAE has given extracts with significantly higher antioxidant capacity compared to other employed extraction methods (MAE > HAE ≥ maceration ≥ UAE, Supplementary Figure S2D).

### 2.7. Antimicrobial Activity of the Extracts

The results of *A. vera* extracts’ antimicrobial activity against two Gram-positive bacteria (*Staphylococcus aureus* and *Enterococcus faecalis*), four Gram-negative bacteria (*Escherichia coli*, *Pseudomonas aeruginosa*, *Klebsiella* spp., and *Proteus* spp.), and one fungus (*Candida albicans*) obtained in disk diffusion method are shown in table of Supplementary Table S3. The antimicrobial activity was assessed by the presence of an inhibition zone.

As can be seen from Supplementary Table S3, *A. vera* leaf waste extracts showed antibacterial activity against both tested Gram-positive strains. Namely, all extracts possessed medium inhibition properties against *S. aureus* (inhibition zone > 10 mm). Against *E. feacalis*, the extracts prepared using HAE and UAE showed weak inhibitory activity (inhibition zone > 5 mm), while macerate and the extract from MAE had very weak activity on the growth inhibition of the mentioned bacteria (inhibition zone < 5 mm). Tested Gram-negative bacteria, as well as *C. albicans* were resistant to all *A. vera* extracts.

### 2.8. The Influence of the Extracts on Cell Viability

**Figure 6:**
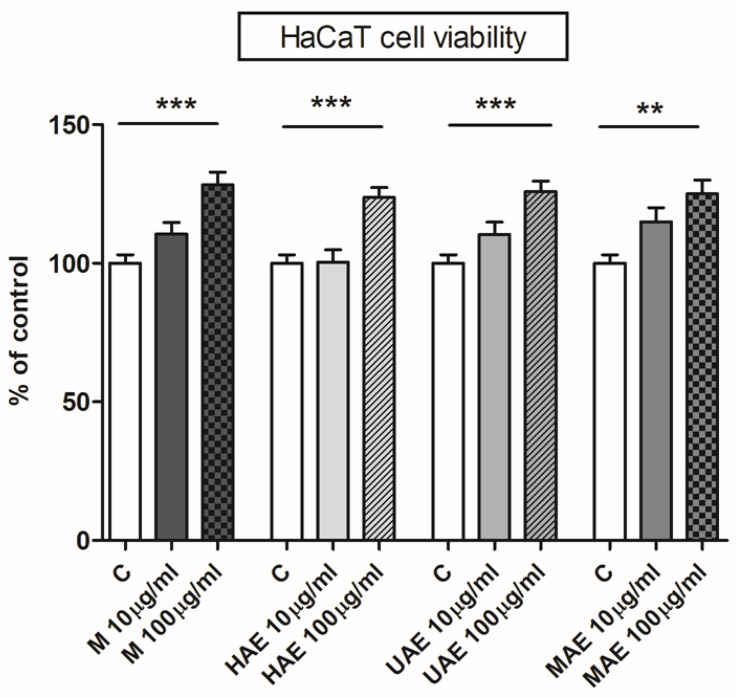
The influence of *Aloe vera* leaf waste extracts prepared using maceration, heat-, ultrasound- and microwave-assisted extractions (M, HAE, UAE, and MAE, respectively) on HaCaT cell (immortalized human keratinocytes) viability; ** *p* < 0.01, *** *p* < 0.001.

Due to their use in traditional medicine and effects on skin cells shown in several studies [1,4], selected *A. vera* leaf waste extracts were examined in terms of skin cell viability. Determination of in vitro cytotoxicity is the first and essential step in the investigation of the potential toxicity of medicinal, pharmaceutical, and cosmetic agents, such as herbal extracts or their bioactive compounds. Therefore, the impact of prepared extracts on HaCaT cell viability was investigated and the results are presented in Figure 6.

Four *A. vera* extracts were subjected to the cytotoxicity assay, and none of them had an unfavorable effect on the cell line’s growth rate, i.e., none of the extracts were cytotoxic (Figure 6). *A. vera* leaf waste extracts had a significant stimulating effect on keratinocytes at concentration of 100 µm/mL (128%, 124%, 126%, and 125% of control for maceration, HAE, UAE, and MAE, respectively).

### 2.9. Physical Properties of the Extracts

Physical properties, including extraction yield (EY), zeta potential (ζ), conductivity (G), density (ρ), surface tension (γ), and viscosity (η) of selected *A. vera* leaf waste extracts (the samples obtained under the optimal extraction conditions in every employed extraction technique for achieving the highest polyphenol content) are presented in Table 5.

The extraction yield was expressed as the dry matter content (%) and the results are presented in Table 5. Regarding the extraction yield, there was a significant difference between the used extraction techniques (MAE > HAE > maceration and UAE). As was expected, the highest content of total extractive substances was measured in the extract prepared using MAE (4.67 ± 0.28%).

The zeta potential of the extracts was measured as the second physical characteristic (Table 5). In the case of *A. vera* extracts, the zeta potential was significantly low (0.14–0.69 mV). The conductivity of the extracts was determined as well (Table 5). The highest conductivity of *A. vera* extracts was measured in the samples from maceration and MAE (1.05 ± 0.05 and 1.00 ± 0.01 mS/cm, respectively), followed by the extract from HAE (0.97 ± 0.01 mS/cm) and UAE (0.92 ± 0.02 mS/cm). Namely, in the case of *A. vera* extracts, the antioxidant capacity determined in DPPH, FRAP, and CUPRAC assays did not correlate with the measured conductivity at all levels (according to the values obtained in DPPH, FRAP, and CUPRAC tests for the mentioned four extracts, 41.08 ± 1.13 µg/mL, 0.195 ± 0.007 mmol/g, and 0.140 ± 0.005 mmol/g for maceration, 41.66 ± 1.76 µg/mL, 0.219 ± 0.003 mmol/g, and 0.132 ± 0.005 mmol/g for HAE, 44.46 ± 2.29 µg/mL, 0.200 ± 0.008 mmol/g, and 0.127 ± 0.002 mmol/g for UAE, and 24.87 ± 1.06 µg/mL, 0.220 ± 0.006 mmol/g, and 0.192 ± 0.003 mmol/g for MAE). On the other hand, determined ABTS radical scavenging antioxidant capacity was in correlation with the measured conductivity and followed a trend: maceration and MAE > HAE > UAE (according to the values obtained in ABTS assay for these four extracts, 2.05 ± 0.11 and 2.30 ± 0.17 µmol TE/g > 1.60 ± 0.13 µmol TE/g > 1.40 ± 0.02 µmol TE/g).

The obtaining of the experimental data on *A. vera* waste extracts’ rheological characteristics, including density, surface tension, and viscosity, is an important task, because of their future application, drying, or encapsulation. Additionally, it is essential to measure the mentioned rheological properties of the extracts because of the evaporation equipment design and evaporation process as well [47].

The results of the density measurement of the extracts are presented in Table 5. The extract prepared using maceration has shown the highest value of density (0.939 ± 0.005 g/mL), while there was no significant difference between the density of other extracts. The obtained results were expected since the extract from maceration was 50% ethanolic, while others were 70% ethanolic (the extracts with the highest TPC according to the results from experimental design, Section 2.2).

As can be seen from Table 5, the highest surface tension was in the extract obtained in maceration (28.7 ± 0.1 mN/m), followed by the extracts from UAE and MAE (27.1 ± 0.2 and 27.0 ± 0.2 mN/m, respectively), while the extract obtained in HAE possessed significantly lower value of surface tension (26.5 ± 0.2 mN/m).

According to the results of the viscosity of the extracts (Table 5), it can be noticed that all extracts possessed low viscosity (3.27–3.45 mPa•s). However, the extracts obtained in maceration and HAE have shown significantly higher viscosity in comparison to UAE and MAE extracts.

## 3. Discussion

In all *A. vera* extracts, a statistically significant increase in polyphenol amount was noticed as the solid-to-solvent ratio increased. It can be explained by the prevention of solvent saturation and the larger diffusion rate, which consequently cause the increase in TPC [28,48,49]. According to Mustafa and Turner [48], high temperature during extraction causes the decrease in the extraction medium viscosity, the improvement of mass transfer and polyphenol recovery, and reaching quick saturation of the medium. Similarly, the differences between low and high examined levels of ratio in UAE can be explained by the mechanism of ultrasound extraction. Namely, ultrasound waves due to acoustic cavitations and thermal effects induce the reduction in the particle size of plant material, damage to the cell walls and membrane, and thus higher polyphenol release and medium saturation [46]. The enhancement of the mass transfer and a higher polyphenol recovery from degraded cells in MAE also result in the rapid saturation of the extraction medium. Hence, the increase in the amount of the extraction medium causes the better release of polyphenol compounds from the herbal matrix [50,51]. According to the literature, a higher amount of plant material induces the rise of viscosity and inhibits the expansion of ultrasound waves; thus, the active part was restricted to a zone located in the vicinity of the ultrasound probe, causing the lower release and diffusion of polyphenol compounds [51,52]. The obtained results are following the observations of Ćujić et al. [25], d’Alessandro et al. [53], and Milutinović et al. [54], where polyphenol yield in chokeberry and yarrow extracts continuously rose with the increment of solid-to-solvent ratio.

Our results are in agreement with the literature data, where the ethanol/water mixture as an extraction medium causes an increase in the solubility of some polyphenols (due to ethanol) and the higher wetting and swelling of the herbal matrix (due to water) [53,55]. In several studies, the water/ethanol mixture was the most efficient solvent for polyphenol extraction [17,27,30,56]. In Vastrad et al.’s study [57], the TPC of *A. vera* water extract was significantly lower in comparison to its ethanol parallel. In HAE, the polarity of the solvent decreases substantially by applying high temperature, making the mixture of water and ethanol the most suitable extraction medium for the extraction of polar, moderately polar, and non-polar polyphenols [48]. Additionally, in UAE, the most suitable solvent for polyphenol extraction was also shown to be an ethanol/water mixture, whereas pure water could not completely extract polyphenols [58]. According to Jovanović et al.’s [19] study, TPC rose in wild thyme extracts prepared using a rising concentration of ethanol (from 0 to 50%) in MAE and then reach a steady state of up to 70% ethanol. Several studies have also reported that the mono-solvent system was not recommended as the adequate medium for polyphenol extraction [30,59,60].

The presented results of the time impact on TPC are in accordance with the literature data and Fick’s second law of diffusion, where the amount of released polyphenols increases with the increase in the extraction time [27,61]. Meterc et al. [62] have also reported that polyphenol content decreased after long extraction from green tea at 80 °C. Laib et al. [63] have also reported that maceration and UAE time had a significant effect on the TPC of *A. vera* leaf extracts. However, prolonged extraction time can cause a decrease in TPC, due to enzymatic degradation, oxidation, and polymerization of polyphenols [31]. In addition, long exposure time in UAE can degrade extracted polyphenols, due to the generation of free radicals by the ultrasound waves [17]. Furthermore, the absence of extraction time influence on TPC in the extracts prepared using HAE can be explained by the occurrence of two stages in the extraction of polyphenol compounds. Namely, there is an initial increase in polyphenol yield at the beginning, the first 15 min, followed by slow extraction during 60 min [25]. Several studies have confirmed that extraction time after 15 min at high temperatures did not have a significant and positive influence on TPC [31,58]. Jovanović et al. [19] have also shown that TPC of wild thyme extracts increased with the increase in the extraction time up to some level (3 min of MAE), and after that started to decrease due to enzymatic degradation or oxidation of polyphenols caused by extended irradiation time.

In HAE, thermal energy increases polyphenol release by disrupting plant cells and their structures and decreases the viscosity of the extraction solvent, which leads to an improved and accelerated extraction process [30,48]. Additionally, the increase in temperature can decrease surface tension, enhancing the wetting of the plant matrix and consequently causing higher efficiency of the extraction [31]. The obtained results are in accordance with the literature data, where higher temperature increased the extraction of polyphenol compounds from different aromatic plants [28,30]. Compared to the other employed extraction techniques in the present study and taking into consideration the industrial requirements, including high extraction yield of the target compounds during short extraction time, HAE can be selected as a suitable procedure for the extraction of polyphenol compounds from *A. vera* leaf waste.

The mentioned differences between the best extraction solvents in various extraction methods can be explained by their mechanisms. Namely, in the case of UAE, there is excessive destruction of plant tissues and cells by ultrasound waves. In specific, created bubbles in the extraction medium provide a rise of temperature and negative pressure and changes on a solid surface, as well as in the cell microstructure, causing the release of cell content and recovery of polyphenols located into different intracellular structures. Additionally, ultrasound waves can break bonds between polyphenols and cell structure molecules, such as proteins, sugars, and lipids, providing a release of different polyphenols compared to maceration extracts [51,52]. The mentioned polyphenols probably show better solubility in ethanol; thus, 70% ethanol extracts possess a higher TPC in comparison to 50% ethanol extracts in UAE. Additionally, according to the literature data, pure ethanol has shown better polyphenol recovery from *A. vera* leaves in comparison to water (~46%) [57]. Although UAE has several advantages over traditional extraction procedures—including the enhancement of extraction efficiency, improvement of the extract quality, and faster kinetics—and over other novel extraction techniques, including lower price and simple operation [64]—in the case of *A. vera* leaf waste, it cannot be recommended as an efficient procedure for the extraction of polyphenol components. The fact that UAE did not provide the extracts with higher polyphenol yield compared to maceration can be explained by the sensitivity of *A. vera* polyphenols that were probably degraded by ultrasound waves and production of free radicals using an ultrasound probe [51,64].

MAE provides higher extraction efficiency and polyphenol yield, and reduced extraction time and solvent consumption, due to the efficient and homogeneous heating of the extraction medium, intercellular superheating, and consequently cell disruption and excessive release of polyphenols [51,64]. However, prolonged extraction time and better extraction yields in MAE do not always provide a higher amount of the target components (e.g., polyphenols) because cell destruction by microwaves can cause the release of a higher quantity of ballast substances, including sugars, proteins, and lipids, as well as the degradation of sensitive polyphenol compounds and a lower yield of non-polar and volatile target components [64,65]. The mean TPC value of all extracts prepared using MAE was not significantly different in comparison to maceration, HAE, and UAE; therefore, MAE cannot be the first choice for the polyphenol extraction from *A. vera* leaf waste.

The obtained values of the flavonoid concentration in *A. vera* extracts are in agreement with the study by López et al. [66], where the content of flavonoids amounted to 1.88 mg per g of the lyophilized *A. vera* leaves. The study by Horžić et al. [16] has shown opposite results, where a higher content of flavonoids from yellow tea was detected in the extracts prepared using UAE compared to the extraction at a high temperature. Additionally, Jovanović et al. [41] reported in their study that wild thyme extracts prepared using high temperature and ultrasound waves possessed higher TFC in comparison to microwaves. However, the explanation of the differences between results lies in the fact that in our study MAE procedure was performed at 100 °C, whereas in the case of wild thyme extraction, the temperature was set at 200 °C which probably caused thermodegradation of flavonoids. Relatively low content of flavonoids determined in all examined *A. vera* extracts is in accordance with the literature data [67], where GC-MS analyses indicated that the majority of the polyphenol components identified in *A. vera* belong to the non-flavonoid group of polyphenols (92%). The obtained results were also confirmed in the LC-MS analysis of our extracts (Section 2.4).

The study by Benzidia et al. [68] reported that the differences in tannin content could be attributed to the used extraction technique and medium, as well as the geographical origin and environmental conditions. Tannins possess biological and pharmacological activities including antioxidative, antibacterial, antiviral, cardioprotective, antitumor, anti-inflammatory, and immunomodulatory [69,70]. Traditional spectrophotometric assays provide fast and straightforward screening for the quantification of various classes of polyphenols in extracts. Nevertheless, due to the complexity of the plant’s secondary metabolites such as polyphenols, and their different reactivity toward assay reagents, a broad spectrum of methods is used for the quantification of the constituents, leading to differing and often non-comparable results [70].

The high temperature present in the microwave reactor leads to the weakening of hydrogen bonds and electrostatic interactions between the polar groups of proteins and the aqueous phase of the membrane, which enables their easier release into the extraction medium [71]. Additionally, most of the proteins together with hydrophobic polyphenols and polysaccharides are found in the cell wall, which is degraded under the influence of microwaves [65,72]. The obtained data of protein content are in agreement with the results of wild thyme extracts, where the highest level of proteins was measured in the extract prepared using MAE [41].

The presented results show that TPC did not correlate strictly with the ABTS antioxidant capacity of the extracts because some other compounds, including sugars, proteins, plant pigments, and free organic acids, have an important role in the neutralization of free radicals [73]. Additionally, the lowest antioxidant activity in the extracts prepared using an ultrasound probe can be explained by the negative influence of ultrasound waves on antioxidant compounds due to the production of free radicals [64]. Similarly, prolonged extraction time in UAE caused a slight decrease in ABTS antioxidant potential of ethanol extracts of *Camellia sinensis* [16] due to polyphenol (flavonoids and anthocyanins) chemical break-down by the ultrasonic treatment [51,74,75].

The obtained results of DPPH antioxidant capacity are in accordance with the literature data, where the lowest DPPH radical scavenging activity of yellow tea extracts prepared using an ultrasound probe was determined after 3 min, while the values were the same after 15 and 30 min [16]. Namely, according to Peanparkdee et al. [76], ultrasound waves prevent an increase in the antioxidant capacity of the extracts from being achieved by lengthening the extraction period after the equilibrium has been achieved due to the potential degradation of sensitive antioxidant compounds, as well as generation of free radicals. Paniwnyk et al. [77] have reported that in the presence of high energy, oxidative reactions can cohabit with the extraction reactions. Since both radical scavenging assays (ABTS and DPPH tests) showed that antioxidant capacity significantly decreased with the decreasing of solid-to-solvent ratio, it can be explained by the diffusion rate of plant antioxidants that is inversely proportional to the viscosity of the extraction medium [78]. Besides that, the better performance of all used extraction techniques (shaking, heat transfer, and propagation of the ultrasound waves and microwaves) can be shown in the dilute medium (at a 1:30 ratio) compared to a more viscous medium (at a 1:10 ratio). According to the studies of Tan et al. [79] and Wong et al. [80], the antioxidant activity of the plant extracts increased as the solvent-to-solid ratio increased as well. Jovanović et al. [81] and Miron et al. [30] have also reported that the antioxidant potential of ethanol extracts of various plants was higher compared to water parallels. Moniruzzaman et al. [82] showed that the flavonoid concentration in ethanol aloe extracts was strongly correlated with DPPH radical scavenging activity and ferric reducing power. As can be seen in Section 2.3, the extract obtained using MAE possesses the highest TFC as well. Jadid et al. [83] have reported that the DPPH radical scavenging activity of the extracts depended, in addition to polyphenol compounds, also on the presence of terpenoids, alkaloids, and saponins. In the study by Tripathi et al. [84], the extracts containing anthraquinones and their glucosides (emodin, aloe-emodin, and emodin glucosides) as major compounds showed concentration-dependent scavenging of DPPH radicals. LC-MS analysis of *A. vera* waste extracts confirmed the presence of the mentioned anthraquinone compounds responsible for the neutralization of free DPPH radicals (Section 2.4).

A significant drop in ferric ion-reducing potential after prolonged extraction time can be explained via the production of free radicals by ultrasound probe during longer extraction and the degradation of plant antioxidants during longer microwave exposure [64]. Prolonged extraction time in MAE, as well as a higher extraction yield, do not mean a large quantity of target antioxidants, because microwaves cause the release of a higher content of ballast compounds, including sugars, lipids, and proteins [64]. This has been previously shown in the case of total proteins of *A. vera* extracts (Section 2.3). The extraction technique significantly affected ferric ion, reducing the capacity of the extracts. FRAP assay is based on the ability of antioxidants, including polyphenol compounds, to reduce Fe^3+^ to Fe^2+^, forming a blue chromophore. Polyphenols present in the extracts, as the donors of electrons, can react with free radicals and stabilize them blocking the chain reactions. According to the literature data, the ferric-reducing ability of *Melissa officinalis* extracts correlates with the polyphenol content of the extract prepared at a high temperature [85]. The discrepancy observed in the comparison of various extraction factors and the results of antioxidant ability determined in different assays is not surprising considering that reagents, targeted antioxidants, mechanism and kinetics of the antioxidant reaction, and conditions during the measurements (pH, wavelength, and time), differ between all used antioxidant tests.

According to Mothapo’s findings [86], a higher solid-to-solvent ratio provides the extracts with a higher cupric ion-reducing activity. Anand et al. [87] have shown that alcoholic extract provided higher ion-reducing potential compared to water extract. The reason for the mentioned differences lies in the various chemical compositions of the extracts. The extraction time had a significant impact on the ion-reducing capacity of the extracts prepared using maceration, UAE, and MAE. Namely, prolonged time induced higher CUPRAC values in the mentioned extraction procedures, but only up to a certain limit, which was in accordance with the TPC as well. Uysal et al. [88] have also reported that prolonged extraction can cause an increase in CUPRAC values. MAE has given extracts with significantly higher antioxidant capacity compared to other employed extraction methods. According to Apak et al. [89], there was a certain correlation between the TPC values measured in the Folin–Ciocalteu method and antioxidant capacity values measured in the CUPRAC assay. Furthermore, the polyphenols that exerted the highest CUPRAC values are catechin, epicatechin, epicatechin gallate, epigallocatechin, epigallocatechin gallate, quercetin, rutin, caffeic, gallic, and chlorogenic acids [86]. However, LC-MS analysis showed the presence of other polyphenol compounds in *A. vera* waste extracts, i.e., anthraquinone derivatives. According to the literature data, anthraquinones, including aloe-emodin, emodin, rhein, chrysophanol, and physcion, are reported to possess antioxidant, anticancer, antiproliferative, and antiangiogenic activity through preventing blood vessel formation in zebrafish embryos, as well as diuretic and laxative properties [90,91]. Nevertheless, the cupric ions can be reduced by various antioxidant compounds apart from polyphenols, including thiols, D-ascorbic acid, mannitol, glucose, etc. [73].

Athiban et al. [92] have reported that ethanolic *A. vera* extract possessed antimicrobial activity against *E. coli* and *S. aureus* (24 mm inhibition zone). According to the literature, *A. vera* can inhibit *S. aureus*, *C. albicans*, *P. aeruginosa*, and *Klebsiella pneumoniae* [90], while gel can enhance the wound healing process in vivo through the elimination of the bacteria that contributed to inflammation [93,94]. However, *Aloe arborescens* ethanol extract did not inhibit the growth of *E. coli* and *C. albicans* strains [95]. Polyphenols, including phenolic acids and flavonoids, are reported to possess antimicrobial activity [93,94]. In comparison to other polyphenol compounds, flavonoids possess a broader spectrum and more potent antibacterial activity due to their ability to neutralize bacterial toxins and inhibit biofilm formation [96,97]. Furthermore, the compounds from plants (the majority having flavonoid structure) are being investigated as potential inhibitors of bacterial efflux systems, which are responsible for the decrease in intracellular concentration of antibiotics and loss of their effectiveness. Thus, the mentioned compounds can make bacteria more susceptible to certain antibiotics. However, according to the results of flavonoid yield, as well as LC-MS analysis of *A. vera* waste extracts (Section 2.3 and Section 2.4), the extracts contained very low levels of flavonoid compounds which can explain their weak antibacterial potential. Additionally, according to the literature data, Gram-positive bacteria are more susceptible to inhibition by herbal extracts compared to Gram-negative bacteria [98].

The potential cytotoxic effect of *A. vera* extracts was studied in vitro on keratinocytes, the key cell type of human skin [99]. HaCaT cells used here are spontaneously immortalized human keratinocytes widely used for in vitro studies. The cell viability assay is based on the reduction of MTT into formazan crystals by viable keratinocytes [100]. The absorbance of dissolved formazan correlates with the number of intact viable cells. The intracellular reducing power is mainly provided by NAD(P)H which is derived from dehydrogenase activity in mitochondria, endoplasmic reticulum, and plasma membrane. The presence of various compounds in herbal extracts has increased the complexity of a screening assay involving cell culture technique [100]. Although cytotoxicity can suggest potential application as an anticancer agent, low toxicity is crucial for the successful development of a pharmaceutical product. According to the literature data, primary and secondary metabolites of plants can serve as new lead compounds for enhancement of wound healing. Namely, polyphenols—such as hydrolysable tannins—act against wound infection and stimulate keratinocyte proliferation, while saccharides induce proliferation of keratinocytes, stimulate formation of collagen and extracellular matrix, and can trigger keratinocytes into differentiation [99,101]. The effects on the cells are in many cases highly specific via receptor-mediated interactions by induction of specific growth factors [99]. According to the results of non-cytotoxic effects of the prepared *A. vera* extracts on keratinocytes, it may be regarded as encouraging because the samples demonstrated a lack of cytotoxicity, i.e., no harmful influence on HaCaT cells with additional benefits by antioxidant and antimicrobial activities previously described. Thus, the extracts can have potential applications in pharmaceutical and cosmetic formulations.

The highest content of total extractive substances was measured in the extract prepared using MAE, due to the mechanism of the mentioned extraction technique. Intracellular superheating and consequently cell degradation cause the increase in the recovery of various components, such as polyphenols, terpenes, alkaloids, sugars, lipids, and proteins, within the extraction surrounding resulting in a higher extraction yield [61]. Hence, a higher extraction yield is not always in correlation with the higher content of the target compounds, and the extraction procedure and extraction time significantly affect the content of total extractive substances. Namely, the duration of the extraction process is a function of the molecular weight of the target compounds and the molecular weight of ballast material. In the case when the molecular weight of the target substances is lower in comparison to the molecular weight of ballast substances, the target molecules will diffuse rapidly, and thus the extraction time will be shorter [52].

The measurement of the zeta potential of plant extracts is important from the aspect of their future application, such as their potential encapsulation into various carriers, and use in water and wastewater treatment. Skaf et al. [102] have reported that the extract’s zeta potential depends on extraction conditions, as well as extracted compounds. Additionally, proteins, carbohydrates, and non-polysaccharide polyelectrolytes in plant extracts display negative zeta potential values and have an important role in the coagulation effect of the extract. According to the literature, coagulation and flocculation processes can destabilize the particles in water and wastewater treatment [103]. However, in the case of *A. vera* extracts, the zeta potential was significantly low; thus, they cannot be used in water and wastewater treatment. Nevertheless, according to the literature, *A. vera* waste-adsorbents (as a powder), including milled, air-dried, and chemically or thermally treated plant material, were used for aquatic pollutants removal [15].

Saad Suliman et al. [104] have reported that conductivity can be used as a predictor of the antioxidant capacity of plant extracts. The obtained values of conductivity are in agreement with the literature data, where Jurinjak Tušek et al. [105] have shown that the conductivity of different aromatic plants’ extracts ranged from 0.65 to 1.90 mS/cm and the extracts with good antioxidant activity possessed a good amount of electrical current conductivity. However, the highest value of conductivity in the extracts can also be related to the increase in the number of ions in a solution, not only to the antioxidant capacity of the extract. Saad Suliman et al. [104] have reported that conductivity is influenced by the presence of extraneous ions and is primarily dependent on the ionic species (chloride, sodium, etc.). Therefore, the analysis of the antioxidant capacity of plant extracts using established antioxidant assays is necessary apart from conductivity measurement.

According to Oroian et al. [106], density is a frequently measured parameter in chemical and biochemical engineering, and in the processes that include fluid flow, heat transfer, and mass transfer. The density of extracts is influenced by the extraction conditions and methods, and it is also correlated to the dry substance mass fraction [47,107]. However, in the case of *A. vera* extracts, there was no correlation between the density and extraction yield, which can be explained by the more dominant influence of the extraction medium in comparison to the influence of dry substance mass fraction.

The surface tension has an important effect on different pharmaceutical processes, such as extraction, tablet coating, preparation of various drug carriers, etc. Fathi-Azarbayjani et al. [108] have reported that extraction efficiency, formulation, and stability of the emulsions or liposomes with plant extracts were significantly affected by surface tension. The highest surface tension was in the extract obtained in maceration, followed by the extracts from UAE and MAE, while the extract obtained in HAE possessed significantly lower value of surface tension. The obtained higher value of surface tension in the extracts from maceration was expected due to the above-mentioned influence of the extraction medium (50% ethanol), as in the case of the density. Namely, a higher volume of water compared to 70% ethanolic extracts leads to the relatively high interaction of water molecules through hydrogen bonds, resulting in a higher value of surface tension [109]. However, the differences in surface tension between the other three extracts with the same extraction solvent (70% ethanol) can be explained by the measured TPC in the mentioned extracts. As the polyphenol concentration in the extracts increases (HAE > UAE and MAE, Table 2), a decrease in surface tension can be noticed (HAE < UAE and MAE, Table 5), which is in agreement with the literature data [109,110,111]. Namely, lower surface tension provides faster penetration of the extraction solvent into the plant matrix and increased matrix–solvent contact surface, allowing further polyphenol release in the extract [110].

The viscosity of plant extracts should be measured to predict potential difficulties that can occur in the use of highly viscous extracts in various food, pharmaceutical, and cosmetic products. All *A. vera* extracts possessed low viscosity. However, the extracts obtained in maceration and HAE have shown significantly higher viscosity, which can be explained by the influence of 50% ethanol as an extraction medium in maceration and a higher polyphenol yield in HAE. The liquid with lower viscosity shows higher diffusivity and thus can easily access the pores of the plant matrix and provide higher extraction efficiency [112]. Despite the low viscosity in *A. vera* extracts prepared using UAE and MAE, no enhancement in the TPC was observed (Table 2). According to Hemwimol et al. [113], when UAE was carried out in the mixture of water/ethanol, despite decreased viscosity, there was no increase in the extraction efficiency that can be explained by the excessive generation of free radicals from the ultrasound dissociation of water. Ruesgas-Ramó et al. [114] have reported that the viscosity was related to the free volume, and thus the increase in surface tension resulted in the decrease in free volume and consequently the increase in the viscosity of the extraction solvent. Mason et al. [112] have reported that liquid with low viscosity also possesses low density. Therefore, these can be the reasons for the lower viscosity of UAE and MAE samples in comparison to the extract obtained in maceration. The obtained values of viscosity are in agreement with the literature data, where the viscosity of *Satureja montana* ethanol extracts was ~2.90 mPa•s [111].

## 4. Materials and Methods

### 4.1. Plant Material, Reagents, and Standards

Fresh and green *A. vera* leaves were separated from the plant and the leaves were subsequently washed using distilled water to clear them of dust and impurities. The aloe gel was carefully removed from the leaves using a razor blade and the leaves were washed again using distilled water. Clean and empty leaves that represent the waste were cut into small pieces of approximately 3 × 3 cm and freeze-dried. Firstly, the leaves were frozen in a freezer at −80 °C for 1 h and subsequently freeze-dried in Beta 2-8 LD plus (Christ, Germany) at −75 °C and pressure of 0.011 mbar for 24 h (main drying) and at −65 °C and pressure of 0.054 mbar for 1 h (final drying). The dried *A. vera* plant material was then ground into a very fine powder using a non-metallic electric grinder (particle size of ~0.3 mm). The plant material was maintained in zipper storage bags in a dry and dark place until future extraction.

Distilled water was purified through a Simplicity UV^®^ water purification system (Merck Millipore, Merck KGaA, Darmstadt, Germany), while water, used for the LC-MS analysis was purified by TKA water purification system (TKA, Niederelbert, Germany). Ethanol (p.a.) was obtained from Fisher Science (Loughborough, UK), while LC-MS grade methanol and formic acid from Sigma-Aldrich (Burlington, MA, USA). Analytical standard emodin was purchased from Fluka (Buchs, Switzerland). For chemical spectrophotometric assays and/or for investigations of biological activities, the following chemicals were used: ethanol and sodium carbonate (Fisher Science, Loughborough, UK), Folin–Ciocalteu reagent, Coomassie^®^ Brilliant blue G 250, phosphoric acid, vanillin, hydrochloric acid, and phosphoric acid (Merck, Rahway, NJ, USA), gallic acid (Merck, Darmstadt, Germany), sodium nitrite (Alkaloid, Skopje, Macedonia), aluminum chloride, 2,2’-azino-bis(3-ethylbenzothiazoline-6-sulphonic acid) or ABTS, 6-hydroxy-2,5,7,8-tetramethylchroman-2-carboxylic acid or Trolox, 2,2-diphenyl-1-picrylhydrazyl or DPPH, 2,4,6-tri-(2-pyridyl)-5-triazine, iron(III) chloride, iron(II) sulfate, copper(II)-chloride, ammonium acetate, neocuproine, methanol, and vanillin (Sigma-Aldrich, Darmstadt, Germany), 1 M sodium hydroxide (Alfapanon, Bački Petrovac, Serbia), catechin (Sigma Chemical, St. Louis, MO, USA), potassium persulfate (Centrohem, Stara Pazova, Serbia), Mueller Hinton Agar Plate and Sabouraud Dextrose Agar Plate (ProMedia, Kikinda, Serbia), phosphate-buffered saline (PBS), thiazolyl blue tetrazolium bromide (MTT), and sodium dodecyl sulfate (SDS). 

HaCaT cells, spontaneously immortalized human keratinocytes, were kindly provided by Charles H. Graham (Queen’s University, Kingston, ON, Canada). Cells were maintained in RPMI 1640 (GIBCO BRL Thermo Scientific, Waltham, MA, USA) supplemented with 10% fetal bovine serum (FBS) and containing 1% antibiotic–antimycotic mixture (Capricorn Scientific, Ebsdorfergrund, Germany), hereafter referred to as complete medium.

### 4.2. Preparation of the Extracts

#### 4.2.1. Maceration

Maceration was carried out in the incubator shaker (KS 4000i control, IKA, Königswinter, Germany) at 25 °C and a speed of 220 rpm, using three solid-to-solvent ratios (1:10, 1:20, and 1:30 g/mL), three types of solvent (50 and 70% *v*/*v* ethanol, and water), and three extraction times (30, 45, and 60 min). The extracts were prepared in an Erlenmeyer flask (50 mL), according to the solid-to-solvent ratios, with 20 mL of the extraction solvent. Namely, 2 g of plant material and 20 mL of the extraction solvent (at a 1:10 ratio), 1 g of the plant material and 20 mL of the solvent (at a 1:20 ratio), and 0.67 g of the plant material and 20 mL of the solvent (at a 1:30 ratio) were used. The flasks were covered with aluminum foil to avoid light exposure and ethanol evaporation.

#### 4.2.2. Heat-Assisted Extraction

HAE was applied for polyphenol extraction using the same factor levels as in the previous case and three extraction times (15, 30, and 45 min) at 80 °C and a speed of 220 rpm in the incubator shaker. The extraction procedure of HAE was the same as for the previously described maceration technique.

#### 4.2.3. Ultrasound-Assisted Extraction

UAE was performed using the ultrasound probe Sonopuls (Bandelin, Berlin, Germany) at 60% amplitude and 25 °C (a flask with the sample was continuously cooled using ice coating during ultrasound extraction and the temperature was measured and controlled). UAE was applied using the same factor levels as in the previous cases and three extraction times (5, 15, and 30 min).

#### 4.2.4. Microwave-Assisted Extraction

MAE was carried out at 100 °C in a microwave reactor, Monowave 300 (Anton Paar, Graz, Austria). According to the solid-to-solvent ratios, a defined amount of plant material was mixed with 10 mL of the extraction medium (because of the technical characteristics of the device), in a closed reactor vial, using a magnetic stirring bar at the speed of 600 rpm. In specific, 1 g of the herbal matrix and 10 mL of the extraction medium (at a 1:10 ratio), 0.5 g of the solid and 10 mL of the solvent (at a 1:20 ratio), and 0.33 g of the solid and 10 mL of the medium (at a 1:30 ratio) were used. MAE was performed using the same factor levels as in the previous cases and three extraction times (1, 2, and 3 min).

All extracts were filtered through a cellulose filter (fine pore, 0.45 µm) and stored at 4 °C until further analyses.

#### 4.2.5. Lyophilization of the Extracts

In order to obtain the samples for LC-MS analysis, liquid extracts (the samples obtained under the optimal extraction conditions in every employed extraction technique for achieving the highest polyphenol content) were dried. Additionally, the samples for FT-IR spectroscopy, antimicrobial, and skin regeneration potential analyses were lyophilized. The ethanol from the extracts was evaporated using Heizbad Hei-VAP (Heidolph, Schwabach, Germany) at 40–50 °C, a pressure of 50 mbar, and a rotation speed of 150 rpm. Subsequently, the sample was frozen in the freezer, at −80 °C for 1 h, and freeze-dried at −75 °C and pressure of 0.011 mbar for 24 h and at −65 °C and pressure of 0.054 mbar for one additional hour (Alpha 2-4 LSCplus, Christ, Osterode am Harz, Germany).

### 4.3. Chemical Characterization of the Extracts

#### 4.3.1. Determination of the Total Polyphenols

The total polyphenol content (TPC) was evaluated spectrophotometrically using the modified Folin–Ciocalteu method [115]. The extract (20 µL) was added to distilled water (1.2 mL) and mixed with Folin–Ciocalteu reagent (100 µL). Subsequently, 20% sodium carbonate solution (300 µL) was added, and the volume was made up to 2 mL with distilled water. The samples were left in the dark at 25 °C for 2 h. The absorbance was measured at 765 nm against a blank. Gallic acid was used as a standard for the calibration curve. The TPC was expressed as milligrams of gallic acid equivalents per g of plant material (mg GAE/g).

#### 4.3.2. Determination of the Total Flavonoids

The total flavonoid content (TFC) in the selected *A. vera* extracts (the samples obtained under the optimal extraction conditions in every employed extraction technique for achieving the highest polyphenol content) was determined by a colorimetric assay described by Xi and Yan [24]. The extract (250 µL) was mixed with 5% sodium nitrite solution (75 µL) and distilled water (1.25 mL). After 5 min of incubation, 8.8% aluminum chloride solution (150 µL) was added. Next, 1 M sodium hydroxide solution (500 µL) was transferred at the 6th min. The mixture was diluted to 3 mL with distilled water. The absorbance was measured at 510 nm against the blank. Catechin monohydrate was used as a standard for the calibration curve. The TFC was expressed as milligrams of catechin equivalents per g of plant material (mg CE/g).

#### 4.3.3. Determination of the Condensed Tannins

The condensed tannin content in the selected *A. vera* extracts (the same samples as in the case of TFC) was determined by the vanillin–HCl method [116]. The extract (200 µL) was pipetted out into a test tube. Then, 4% vanillin in methanol (1.5 mL) and concentrated HCl (750 μL) were added and incubated for 15 min. The absorbance was measured at 500 nm. Catechin monohydrate was used as a standard for the calibration curve, and condensed tannins were expressed as milligrams of CE per g of plant material (mg CE/g).

#### 4.3.4. Determination of the Total Proteins

The total protein content in the selected *A. vera* extracts (the same samples as in the case of TFC) was analyzed by using a standard test for the determination of total proteins defined by Bradford [117]. The Bradford dye reagent was prepared according to the following protocol: Coomassie brilliant blue G-250 (100 mg) was dissolved in ethanol (50 mL) and phosphoric acid (85%, *v*/*v*, 100 mL) was added. Subsequently, the stock solution was made up to 1 L using distilled water. The assay was performed using 50 μL of the extract and 2.5 mL of Bradford dye reagent and the absorbance was measured at 595 nm. Albumin was used as a standard for the calibration curve. The results were expressed as mg of proteins per g of plant material (mg/g).

#### 4.3.5. LC-MS Analysis

LC-MS analysis of the selected *A. vera* extracts (the samples obtained under the optimal extraction conditions in every employed extraction technique for achieving the highest polyphenol content) was performed using an Agilent LC/MS System 1260/6130 (Agilent Technologies, Waldbronn, Germany), equipped with ChemStation software Rev. B.04.03-SP1, degasser (model G1311B), quaternary pump (G1311B/1260), autosampler (G1329B), fraction collector (G1364C), diode-array detector (DAD) (G4212B), single quadrupole API-ESI MSD (6130), and active splitter (G1968D), using Zorbax SB-Aq column (150 × 3.0 mm; 3.5 μm particle size, Agilent Technologies), operating at 25 °C. The mobile phases consisted of 0.1% (*v*/*v*) formic acid in water (phase A) and methanol (phase B). The gradient program was as follows: 10% to 90% B (40 min). The flow rate was 0.35 mL/min, and the injection volume of 2 μL. DAD was operating at 210 and 280 nm. ESI mass spectra (MS) were recorded in the negative ion mode: in the full-scan mode in the range of *m*/*z* 80–2500. Optimized ion source parameters were as follows: fragmentor voltage of 250 V and 100 V in full-scan, and 100 V in SIM mode, nebulizing drying gas flow of 10 L/min at 350 °C, nebulizer pressure of 40 psi, and capillary voltage of 3500 V.

The structure of the detected compounds was characterized to the highest reliable level analyzing their UV or MS spectra. The peak areas obtained by DAD (at 210 nm) were used for the quantitative analysis by the external standard method. Calibration curves were obtained using seven different concentrations of emodin as commercial standard compound. The data for each concentration were recorded three times. Linearity and linear ranges, and limits of detection (LOD) and quantification (LOQ) for the compounds were determined according to International Conference on Harmonization guidelines (ICH, 2005) [118]. Namely, obtained regression equations were used to test linearity by the calculation of correlation coefficients (r2), as well as to determine LOD and LOQ using the standard deviations of the response (σ) and the calibration curve slope (a), in the following way:

LOD = 3.3 × σ/a(1)

LOQ = 10 × σ/a(2)

Calibration curve regression equation for emodin was y = 13,655.7800x−59.7597, r^2^ was 1.0000, in a linear range of 0.03–1.75 μg/mL. LOD and LOQ of emodin was 0.032 and 0.094 μg/mL, respectively.

#### 4.3.6. FT-IR Spectroscopy

FT-IR spectra of the selected lyophilized *A. vera* extracts (the same samples as in the case of HPLC analysis) were recorded in the transmission mode between 400 and 4000 cm^−1^ using a Nicolet iS10 spectrometer (Thermo Scientific, Stockholm, Sweden).

### 4.4. Antioxidant Capacity of the Extracts

The antioxidant capacity of all prepared *A. vera* leaf waste extracts was examined using four antioxidant assays: ABTS, DPPH, ferric reducing antioxidant power (FRAP), and cupric reducing antioxidant capacity (CUPRAC) tests.

#### 4.4.1. ABTS Assay

The ABTS assay is based on the reduction in ABTS^•+^ free radicals by antioxidant compounds from the sample [119]. A mixture of ABTS solution (5 mL) and potassium persulfate solution (88 µL) was left to react for 24 h at 4 °C. The ABTS^•+^ working solution was diluted using ethanol (an absorbance of ~0.700 at 734 nm). ABTS^•+^ solution (2 mL) was mixed with diluted liquid extract (1:9, 20 µL). After 6 min of incubation, the absorbance was measured, and the radical scavenging activity of the extract was calculated using the following equation:

∆A = A_0_ − A_x_ (3)

where A_0_ was the absorbance of ABTS^•+^ solution, while A_x_ was the absorbance of ABTS^•+^ solution and the extract. Trolox was used as a standard for the calibration curve. The scavenging capacity was expressed as µmol Trolox equivalents per g of plant material (µmol TE/g).

#### 4.4.2. DPPH Assay

The antioxidant capacity of the extracts was also measured via hydrogen donating or radical scavenging ability using the stable DPPH· radicals [24]. Various concentrations of liquid extract (200 μL) were mixed with 2.8 mL of ethanol DPPH^•^ radical solution (an absorbance of ~0.800 at 517 nm). The absorbance was recorded after 20 min of incubation and the percentage of inhibition was calculated using the following equation:

% inhibition = (A_0_-A_x_) × 100/A_0_ (4)

where A_0_ was the absorbance of the control and A_x_ was the absorbance of DPPH^•^ solution and extract. The results were expressed as IC_50_ (mg/mL) which represented the concentration of the extract required to neutralize 50% of DPPH^•^ radicals.

#### 4.4.3. FRAP Assay

The ferric-reducing antioxidant power of all prepared *A. vera* extracts was determined as well. The FRAP reagent was prepared according to the protocol given by Guo et al. [120]. Namely, 2.5 mL of a 10 mmol/L 2,4,6-tri-(2-pyridyl)-5-triazine solution in 40 mmol/L HCl, 2.5 mL of 20 mmol/L FeCl_3_ and 25 mL of 0.3 mol/L acetate buffer (pH 3.6) was prepared and warmed at 37 °C. The extract (40 µL) was mixed with distilled water (0.2 mL) and FRAP reagent (1.8 mL). The absorbance was measured at 593 nm after 10 min of incubation at 37 °C. FeSO_4_ was used as a standard for the calibration curve. The results were expressed as the concentration of antioxidant compounds having a ferric-reducing ability equivalent to that of 0.5 mmol/L of FeSO_4_.

#### 4.4.4. CUPRAC Assay

The cupric ion-reducing antioxidant activity was determined according to the procedure of Petrović et al. [121] with a slight modification. The solution of cupric(II) ion (10^−2^ mol/mL) was prepared by dissolving 0.0853 g of the copper (II)-chloride dihydrate into 250 mL of distilled water. Ammonium-acetate buffer solution (1 mol/mL) was prepared by dissolving 19.27 g of ammonium acetate in 250 mL of distilled water. The fresh solution of neocuproine was prepared by dissolving 0.078 g of neocuproine in 50 mL of methanol (7.5·10^−3^ mol/mL). Each reaction solution consisted of 0.8 mL of the extract, 1 mL of copper (II)-chloride solution, 1.2 mL of ammonium-acetate buffer solution, and 1 mL of neocuproine solution. The absorbance was measured at 450 nm after incubation for 30 min in the dark place. Trolox was used as a standard for the calibration curve. The results were expressed as mmol of Trolox equivalents per g of plant material (mmol TE/g).

All absorbance readings were performed using the UV Spectrophotometer UV-1800 (Shimadzu, Kyoto, Japan). Every spectrophotometric measurement was conducted in triplicate.

### 4.5. Antimicrobial Analysis

The antimicrobial potential of the selected lyophilized *A. vera* extracts (the samples obtained under the optimal extraction conditions in every employed extraction technique for achieving the highest polyphenol content) was investigated using two Gram-positive bacteria (*S. aureus* and *E. feacalis*), four Gram-negative bacteria (*E. coli*—resistant on ampicillin, amoxicillin, ciprofloxacin, trimethoprim, and fosfomycin, intermediate sensitive on amoxicillin and clavulanic acid, and sensitive on cephalexin, ceftriaxone, gentamicin, amikacin, and nitrofurantoin, *P. aeruginosa*, *Klebsiella* spp., and *Proteus* spp.), and one fungus (*C. albicans*) all isolated from clinical samples in the microbiology laboratory of the Institute for the Application of Nuclear Energy, INEP, Belgrade, Serbia. The antimicrobial activity was examined using the disk diffusion method [122]. Cultivation of microorganisms was performed aerobically for 24 h on Blood agar (for bacteria) and 48 h on Sabouraud agar (for fungus). The microorganism was incubated at 37 °C. The inoculum was prepared to an optical density of 0.5 McFarland (1.5 × 10^8^ CFU/mL) and the inoculum was spread on Petri dishes with Mueller Hinton agar (for bacteria) or Sabouraud agar (for fungus). Subsequently, 60 μL of the dissolved lyophilized extracts (175 mg/mL) were transformed into the wells made in agar and incubated aerobically for 24 h at 37 °C. The criterion for detecting inhibitory activity was the inhibition zone diameter (inhibition zone diameter < 5 mm—very weak inhibitory activity, >5 mm—weak inhibitory activity, >10 mm—medium inhibition, and >15 mm—very strong inhibitory activity). Each test was performed in triplicate. The previously mentioned antibiotics and fluconazole were used in parallel experiments to control the sensitivity of the test bacterial and fungal strains, respectively.

### 4.6. Cell Viability Assay

The viability of HaCaT cells, spontaneously immortalized human keratinocytes, was assessed using the MTT assay [123]. The cells (2 × 104/well) were seeded in 96-well plates in 100 µL of the complete medium and allowed to adhere overnight at 37 °C in a humidified incubator with 5% CO_2_. After 24 h of incubation, cells were rinsed with warm, sterile PBS and further cultured in complete medium with control or *A. vera* extracts at 10 and 100 µg/mL. The cells were incubated for 24 h at 37 °C. Upon treatment, medium was removed, and cells were rinsed with PBS. Then, 100 μL of PBS (10% PBS) containing MTT in final concentration of 0.5 mg/mL added and cells were incubated for 2 h at 37 °C, 5% CO_2_. Subsequently, 100 μL of 10% SDS (0.01 N HCl) was added to each well and the plate was further incubated at 37 °C overnight to ensure complete solubilization of formazan crystals. After 24 h, absorbance was measured at 570 nm using a microplate reader (ELx800, BioTek, Winooski, VT, USA).

### 4.7. Physical Characterization of the Extracts

#### 4.7.1. Extraction Yield

The extraction yield was expressed as the dry matter content of the selected *A. vera* extracts (the samples obtained under the optimal extraction conditions in every employed extraction technique for achieving the highest polyphenol content) and calculated using the following equation:

dry matter content = 100- ((a-b) × 100)/m(5)

where a represents the weight of the vessel containing the extract before drying (g), b represents the weight of the vessel containing the extract after drying at 105 °C to constant mass, and m represents the weight of the extract. Dry matter content is expressed as %.

#### 4.7.2. Zeta Potential and Conductivity Analyses

The zeta potential and conductivity of the selected *A. vera* extracts (the same samples as in the case of the extraction yield) were determined using photon correlation spectroscopy in Zetasizer Nano Series, Nano ZS (Malvern Instruments Ltd., Malvern, UK). Each extract was measured three times at 25 °C.

#### 4.7.3. Density, Surface Tension, and Viscosity Analyses

The density and surface tension of the selected *A. vera* extracts (the same samples as in the case of the extraction yield) were determined using silicon crystal as the immersion body and Wilhelmy plate, respectively, in Force Tensiometer K20 (Kruss, Hamburg, Germany). Each extract (20 mL) was examined three times at 25 °C.

The viscosity of the selected *A. vera* extracts (the same samples as in the case of the extraction yield) was examined using Rotavisc lo-vi device equipment with VOL-C-RTD chamber, VOLS-1 adapter, and spindle (IKA, Königswinter, Germany). Each extract (6.7 mL) was examined three times at 25 °C.

### 4.8. Statistical Analysis

The statistical analysis was performed by using the analysis of variance (one-way ANOVA) followed by Duncan’s post hoc test, and 2^3^ full factorial design within the statistical software STATISTICA 7.0. The differences were considered statistically significant at *p* < 0.05, *n* = 3.

#### 4.8.1. Screening of Factors’ Influence

In the screening analysis (as the first step in the statistical processing of data), the selection of the levels of each examined factor (solid-to-solvent ratio, solvent type, and extraction time) that influence the TPC has been performed. Statistical significance between factor levels has been estimated on triplicate samples through one-way ANOVA followed by Duncan’s post hoc test at *p* < 0.05 level. Therefore, means ± standard deviation followed by different letters in charts and tables differs significantly. Selected two levels of each of the three examined extraction factors which provided the highest polyphenol content were subjected to 2^3^ factorial design. One-way ANOVA and Duncan’s post hoc test were also used for the examination of factor and extraction technique influence on the antioxidant potential of the extracts, as well as the impact of extraction technique on all other examined parameters.

#### 4.8.2. Factorial Design

The factorial design method was used for the optimization of the extraction conditions (factors), i.e., independent variables. Namely, 2^3^ full factorial design was employed to investigate the impact and choose the optimum level of solid-to-solvent ratio (1), solvent type (2), and extraction time (3), as independent variables for achieving the highest polyphenol content, as the dependent variable. Each factor was examined at the two most promising levels using the upper and lower limits chosen based on the previously mentioned screening analysis (Section 4.8.1, one-way ANOVA and Duncan’s post hoc test).

#### 4.8.3. Statistical Analysis of Results of the LC-MS Method

The comparison of anthraquinone composition among selected *A. vera* leaf waste extracts was conducted using multivariate statistical analysis according Milutinović et al. [124]. Principal component analysis (PCA) was used to find compounds that contribute to the variation and differences between investigated extracts. Coded values of the absolute content (the percentages of the detected compounds or g/100 g of dried extract) produced the best output ordination in PCA based on Bray–Curtis pair-wise distance matrix. The analyses were calculated with STATISTICA 7.0. software (Statsoft Inc., Tulsa, OK, USA).

## 5. Conclusions

This study aimed to evaluate the effects of different solid-to-solvent ratios, solvent types, and extraction times on the extraction of polyphenols from *A. vera* leaf waste using various extraction methods (maceration, HAE, UAE, and MAE). The results indicated that the solid-to-solvent ratio, solvent type, extraction time (except in HAE), and extraction technique significantly affected the TPC. According to TPC, the efficiency of the extraction technique for all variables is ranked by significance in the following order: HAE ≥ MAE ≥ maceration and UAE. Concerning the experimental design, the optimal extraction conditions and technique for achieving the highest TPC are the solid-to-solvent ratio of 1:30, 70% ethanol as an extraction medium, and 30 min of HAE. Total flavonoid and protein contents were significantly higher in the extract prepared using MAE, followed by HAE, while total condensed tannin content was the highest in HAE. Furthermore, by LC-MS analysis overall 13 anthraquinone and chromone derivatives were identified and quantified. FT-IR spectra showed the presence of functional groups of bioactive compounds, while the variations in the FT-IR spectra of the extracts obtained by different extraction procedures are relatively minor. The influence of extraction conditions on the antioxidant ability of the extracts depended on applied antioxidant assays. The extracts possessed medium inhibition properties against *S. aureus* and weak inhibitory activity against *E. feacalis*, whereas all tested Gram-negative bacteria and *C. albicans* were resistant. *A. vera* extracts had no harmful influence on HaCaT cells and showed a significant stimulating effect on keratinocytes at a higher concentration. Regarding the extraction yield, there was a significant difference between the used extraction techniques (MAE > HAE > maceration and UAE). Conductivity and zeta potential values were significantly low, while the density, surface tension, and viscosity of the extracts had adequate values for the potential drying or encapsulation processes. The presented study is an initial step in the production of polyphenol-rich extracts from *A. vera* waste aimed to be used for the potential preparation of pharmaceutical and cosmetic formulations for the skin. Further research should be concerned with the encapsulation of selected extracts for improving their stability and bioavailability and providing controlled release of the active compounds. 

## Figures and Tables

**Table 1:** The impact of each factor levels on total polyphenol content (TPC) in *Aloe vera* leaf waste extracts.

		TPC [mg GAE/g of Plant Material]							
Factor	Level	Extraction Techniques							
		Maceration		HAE		UAE		MAE	
Solid-to- solvent ratio [g/mL]	1:10	5.23 ± 0.70 ^c,^*		6.61 ± 0.47 ^c^		3.51 ± 0.19 ^b^		6.22 ± 0.51 ^c^	
	1:20	7.14 ± 0.53 ^b^		8.18 ± 0.28 ^b^		6.29 ± 0.47 ^a^		7.33 ± 0.27 ^b^	
	1:30	9.95 ± 0.51 ^a^		9.76 ± 0.27 ^a^		6.74 ± 0.14 ^a^		8.39 ± 0.65 ^a^	
Solvent type	50% Et-OH	9.47 ± 0.78 ^a^		9.32 ± 0.79 ^b^		6.95 ± 0.23 ^b^		9.08 ± 0.71 ^a^	
	70% Et-OH	6.96 ± 0.24 ^b^		10.40 ± 0.28 ^a^		9.24 ± 0.34 ^a^		9.05 ± 0.93 ^a^	
	water	6.07 ± 0.18 ^c^		7.86 ± 0.08 ^c^		5.22 ± 0.17 ^c^		7.61 ± 0.62 ^b^	
Time ** [min]		30	8.31 ± 0.72 ^b^	15	9.57 ± 0.72 ^a^	5	7.44 ± 0.12 ^b^	1	7.64 ± 0.40 ^b^
		45	9.66 ± 0.51 ^a^	30	9.86 ± 0.91 ^a^	15	8.03 ± 0.23 ^a^	2	8.70 ± 0.42 ^a^
		60	8.43 ± 0.38 ^b^	45	9.95 ± 0.70 ^a^	30	8.07 ± 0.24 ^a^	3	8.82 ± 0.64 ^a^

* Values with the same letter in each column group (for every examined factor) showed no statistically significant difference (*p* > 0.05; *n* = 3; analysis of variance, Duncan’s post hoc test); all levels of one factor were combined with all levels of another two factors; ** extraction time varied depending on the extraction techniques; GAE, gallic acid equivalents; HAE, heat-assisted extraction; UAE, ultrasound-assisted extraction; MAE, microwave-assisted extraction.

**Table 2:** Experimental design for screening of factors’ influence on total polyphenols content (TPC) in *Aloe vera* leaf waste extracts prepared using maceration, heat-, ultrasound-, and microwave-assisted extractions (HAE, UAE, and MAE, respectively), with the observed and predicted values.

S:S Ratio *	Solvent Type	Time	S:S Ratio [g/mL]	Solvent Type	Time [min]	TPC [mg GAE/g]	
						Maceration	
Design			Factor Levels			Observed	Predicted
−1	−1	−1	1:20	50% Et-OH	30	4.99 ± 0.02	5.14
−1	1	−1	1:20	70% Et-OH	30	3.79 ± 0.16	3.65
**1**	**−1**	**1**	**1:30**	**50% Et-OH**	**45**	**10.09 ± 0.18**	**10.11**
1	1	1	1:30	70% Et-OH	45	6.96 ± 0.03	6.81
−1	−1	1	1:20	50% Et-OH	45	7.15 ± 0.45	7.00
−1	1	1	1:20	70% Et-OH	45	5.20 ± 0.20	5.35
1	−1	−1	1:30	50% Et-OH	30	8.31 ± 0.51	8.17
1	1	−1	1:30	70% Et-OH	30	4.84 ± 0.18	4.99
						**HAE**	
						**Observed**	**Predicted**
−1	−1	−1	1:20	50% Et-OH	15	8.11 ± 0.09	8.15
−1	−1	1	1:20	70% Et-OH	15	9.33 ± 0.15	9.29
−1	1	−1	1:30	50% Et-OH	30	9.52 ± 0.50	9.34
**−1**	**1**	**1**	**1:30**	**70% Et-OH**	**30**	**10.42 ± 0.10**	**10.35**
1	−1	−1	1:20	50% Et-OH	30	8.19 ± 0.20	8.14
1	−1	1	1:20	70% Et-OH	30	9.57 ± 0.15	9.61
1	1	−1	1:30	50% Et-OH	15	9.29 ± 0.13	9.48
1	1	1	1:30	70% Et-OH	15	10.10 ± 0.36	10.15
						UAE	
						Observed	Predicted
−1	−1	−1	1:20	50% Et-OH	5	4.76 ± 0.25	4.67
−1	1	−1	1:20	70% Et-OH	5	6.43 ± 0.32	6.52
1	−1	1	1:30	50% Et-OH	15	8.00 ± 0.08	7.94
**1**	**1**	**1**	**1:30**	**70% Et-OH**	**15**	**9.24 ± 0.24**	**9.33**
−1	−1	1	1:20	50% Et-OH	15	6.28 ± 0.33	6.37
−1	1	1	1:20	70% Et-OH	15	9.10 ± 0.15	9.02
1	−1	−1	1:30	50% Et-OH	5	7.49 ± 0.11	7.53
1	1	−1	1:30	70% Et-OH	5	8.21 ± 0.26	8.12
						MAE	
						Observed	Predicted
−1	−1	−1	1:20	50% Et-OH	1	6.40 ± 0.23	6.39
−1	−1	1	1:20	70% Et-OH	1	6.73 ± 0.21	6.74
−1	1	−1	1:30	50% Et-OH	2	8.72 ± 0.30	8.70
**−1**	**1**	**1**	**1:30**	**70% Et-OH**	**2**	**9.00 ± 0.66**	**9.03**
1	−1	−1	1:20	50% Et-OH	2	7.35 ± 0.19	7.33
1	−1	1	1:20	70% Et-OH	2	7.47 ± 0.35	7.46
1	1	−1	1:30	50% Et-OH	1	7.65 ± 0.28	7.64
1	1	1	1:30	70% Et-OH	1	8.25 ± 0.27	8.20

* S:S, solid-to-solvent; GAE, gallic acid equivalent; bold values represent the combination of the extraction factors’ level for achieving the highest polyphenol content.

**Table 3:** MS spectral data of the detected constituents of *Aloe vera* leaf waste extracts.

	R_t_ (min)	*m*/*z* [M-H]^−^ (100 V)	*m*/*z* (250 V)	Compound
**1**	4.75	463.1	463.1 (5) ^a^, 120.1 (12), 105.1 (100)	Unknown
**2**	8.99	447.1	447.1 (3), 243.1 (4), 188.1 (10), 143.1 (100), 130.1 (25), 117.1 (82), 105 (9)	Hydroxymethyl aloin
**3**	15.29	477.0	477.0 (2), 393.1 (89), 303.0 (10), 285.0 (4), 273.1 (100), 255.1 (6), 245.1 (18), 231.0 (11), 203.1 (8), 191.0 (2), 173.0 (2), 161.1 (1)	Malonylaloesin
**4**	15.99	813.1	813.1 (10), 563.0 (4), 547 (4), 496 (5), 479.0 (10), 395.1 (100), 351.1 (59), 315.0 (5), 305.1 (10), 275.0 (99), 247.1 (11), 231.1 (65), 203.1 (37), 190.1 (2), 185.0 (2), 161.0 (2)	Aloin hexoside derivative
**5**	18.65	593.1	593.1 (100), 473.1 (68), 431.1 (29), 311.0 (32), 296.0 (16), 253.0 (12), 175.0 (8)	Nataloin derivative
**6**	20.51	813.1	813.1 (20), 791.1 (17), 770.1 (26), 769.0 (82), 743.0 (15), 735.0 (17), 581.1 (37), 579.1 (13), 565.0 (16), 543.0 (14), 493.1 (16), 433.1 (93), 417.0 (36), 327.0 (41), 313.0 (41), 284.0 (22), 270.0 (100), 255.1 (11), 241.0 (11), 175.0 (26)	Aloin hexoside derivative
**7**	20.92	579.1	579.1 (100), 501.0 (3), 549.1 (39), 417.1 (10), 296.0 (12), 267.0 (5), 255.0 (3), 253.1 (2), 175.0 (2)	Aloin hexoside
**8**	21.54	593.1	593.1 (100), 579.1 (5), 565.0 (4), 459.1 (7), 443.1 (8), 393.0 (5), 325.1 (3), 305.0 (3), 192.0 (3)	Aloe-emodin dihexoside
**9**	22.28	569.1	569.1 (13), 459.1 (9), 417.1 (4), 393.0 (8), 375.0 (100), 325.0 (5), 297.0 (7), 285.0 (4), 255.0 (8), 193.1 (27), 175.0 (3), 134.1 (7)	Aloeresin
**10**	23.16	417.0	417.0 (9), 325.0 (3), 297.1 (100), 269.1 (3), 255.0 (4), 251.1 (3), 239.0 (1), 207.1 (2)	Aloin B
**11**	23.38	417.1	417.1 (16), 325.1 (2), 297.1 (100), 255.0 (4), 251.0 (2), 225.1 (1)	Aloin A
**12**	23.76	459.0	459.0 (20), 399.1 (4), 339.1 (2), 325.0 (2), 297.1 (100), 255.1 (2), 251.0 (2), 239.0 (1)	Malonylnataloin
**13**	24.33	583.1	583.1 (13), 518.1 (2), 504.1 (2), 481.1 (3), 459.1 (20), 431.1 (7), 399.1 (3), 375.1 (72), 311.1 (13), 297.1 (100), 285.0 (2), 269.1 (13), 255.1 (8), 253.1 (31), 225.0 (6), 207.1 (17)	Aloe-emodin hexoside derivative

^a^ Figures in brackets refer to the relative abundances of the ions.

**Table 4:** The content of detected compounds (1–13) in selected *Aloe vera* leaf waste extracts prepared using maceration, heat-, ultrasound- and microwave-assisted extractions (HAE, UAE, and MAE, respectively) determined in LC-MS analysis.

Compounds	Extracts			
	Maceration	HAE	UAE	MAE
	Content (%, g of Emodin/100 g of Dried Extract)			
1 *	0.085 ± 0.001 **	0.168 ± 0.009	0.095 ± 0.001	0.199 ± 0.000
**2**	0.114 ± 0.000	0.239 ± 0.010	0.199 ± 0.003	0.277 ± 0.011
**3**	0.188 ± 0.005	0.433 ± 0.011	0.489 ± 0.009	0.434 ± 0.008
**4**	0.032 ± 0.000	0.068 ± 0.003	0.022 ± 0.000	0.038 ± 0.000
**5**	0.001 ± 0.000	0.001 ± 0.000	0.006 ± 0.000	0.028 ± 0.006
**6**	0.009 ± 0.000	0.020 ± 0.002	0.013 ± 0.000	0.001 ± 0.000
**7**	0.027 ± 0.001	0.022 ± 0.001	0.005 ± 0.000	0.042 ± 0.003
**8**	0.005 ± 0.000	0.001 ± 0.000	0.029 ± 0.002	0.023 ± 0.000
**9**	0.018 ± 0.001	0.005 ± 0.000	0.059 ± 0.004	0.096 ± 0.005
**10**	0.008 ± 0.000	0.068 ± 0.002	0.039 ± 0.001	0.062 ± 0.002
**11**	0.001 ± 0.000	0.062 ± 0.003	0.024 ± 0.000	0.023 ± 0.000
**12**	0.009 ± 0.000	0.041 ± 0.001	0.050 ± 0.001	0.044 ± 0.001
**13**	0.052 ± 0.000	0.128 ± 0.004	0.181 ± 0.006	0.120 ± 0.002

* 1, unknown compound; 2, hydroxymethyl aloin; 3, malonylaloesin; 4, aloin hexoside derivative; 5, nataloin derivative; 6, aloin hexoside derivative; 7, aloin hexoside; 8, aloe-emodin dihexoside; 9, aloeresin; 10, aloin B; 11, aloin A; 12, malonylnataloin; 13, aloe-emodin hexoside derivative. ** The results represent the means of three determinations ± standard deviation (SD).

**Table 5:** The extraction yield (EY), zeta potential (*ζ*), conductivity (*G*), density (*ρ*), surface tension (*γ*), and viscosity (*η*) of selected *Aloe vera* leaf waste extracts prepared using maceration, heat-, ultrasound- and microwave-assisted extractions (HAE, UAE, and MAE, respectively).

Extraction Method	EY (%)	*ζ* [mV]	*G* [mS/cm]	*ρ* [g/mL]	*γ* [mN/m]	*η* [mPa·s]
maceration	3.85 ± 0.15 ^c,^*	0.14 ± 0.06 ^a^	1.05 ± 0.05 ^a^	0.939 ± 0.005 ^a^	28.7 ± 0.1 ^a^	3.45 ± 0.02 ^a^
HAE	4.20 ± 0.09 ^b^	0.49 ± 0.05 ^a^	0.97 ± 0.01 ^b^	0.916 ± 0.006 ^b^	26.5 ± 0.2 ^c^	3.43 ± 0.02 ^a^
UAE	3.70 ± 0.12 ^c^	0.69 ± 0.06 ^a^	0.92 ± 0.02 ^c^	0.917 ± 0.004 ^b^	27.1 ± 0.2 ^b^	3.28 ± 0.03 ^b^
MAE	4.67 ± 0.28 ^a^	0.28 ± 0.08 ^a^	1.00 ± 0.01 ^a^	0.911 ± 0.007 ^b^	27.0 ± 0.1 ^b^	3.27 ± 0.03 ^b^

* Values with the same letter in each column showed no statistically significant difference (*p* > 0.05; *n* = 3; analysis of variance, Duncan’s post hoc test).

## Data Availability

The datasets generated during and/or analyzed during the current study are available from the corresponding author on reasonable request.

## Supplementary Materials

The following supporting information can be downloaded at: https://www.mdpi.com/article/10.3390/plants12142744/s1. Table S1: Statistical analysis of optimization of maceration, heat-, ultrasound-, and microwave-assisted extractions (HAE, UAE, and MAE, respectively) from *Aloe vera* leaf waste using 2^3^ factorial design; Figure S1: The influence of solid-to-solvent ratio and solvent type on (A) ABTS and (B) DPPH radical scavenging activity, (C) ferric reducing antioxidant power (FRAP), and (D) cupric ion-reducing antioxidant capacity (CUPRAC) of *Aloe vera* leaf waste extracts obtained in maceration, heat-, ultrasound-, and microwave-assisted extractions (HAE, UAE, and MAE, respectively); Table S2: The influence of extraction time on ABTS and DPPH radical scavenging activity, ferric reducing antioxidant power (FRAP), and cupric ion-reducing antioxidant capacity (CUPRAC) of *Aloe vera* leaf waste extracts obtained in maceration, heat-, ultrasound-, and microwave-assisted extractions (HAE, UAE, and MAE, respectively; Figure S2: Effects of extraction techniques on (A) ABTS and (B) DPPH radical scavenging activity, (C) ferric reducing antioxidant power (FRAP), and (D) cupric ion-reducing antioxidant capacity (CUPRAC) of all prepared *Aloe vera* leaf waste extracts using maceration, heat-, ultrasound-, and microwave-assisted extractions (HAE, UAE, and MAE, respectively); different letters represent different population groups based on Duncan’s post hoc test (*p* < 0.05); Figure S3a: Chromatograms of selected *Aloe vera* leaf waste extracts prepared using maceration, heat-, ultrasound- and microwave-assisted extractions (M, HAE, UAE, and MAE, respectively); 1, unknown compound; 2, hydroxymethyl aloin; 3, malonylaloesin; 4, aloin hexoside derivative; 5, nataloin derivative; 6, aloin hexoside derivative; 7, aloin hexoside; 8, aloe-emodin dihexoside; 9, aloeresin, 10 aloin B; 11, aloin A; 12, malonylnataloin; 13, aloe-emodin hexoside derivative; Figure S3b: Structure of the compounds identified in the selected *Aloe vera* leaf waste extracts using LC-MS analysis; Table S3: The antimicrobial activity of selected *Aloe vera* leaf waste extracts prepared using maceration, heat-, ultrasound- and microwave-assisted extractions (HAE, UAE, and MAE, respectively) determined using disk diffusion method.
