# Supplementary material for: From Aloe vera Leaf Waste to the Extracts with Biological Potential: Optimization of the Extractions, Physicochemical Characterization, and Biological Activities
Source: Plants (Basel). 2023 Jul 24;12(14):2744. doi: 10.3390/plants12142744 (PMC10386512; doi:10.3390/plants12142744)
Supplement: Supplementary file 1 [file plants-12-02744-s001.zip › Figure S1.pdf]

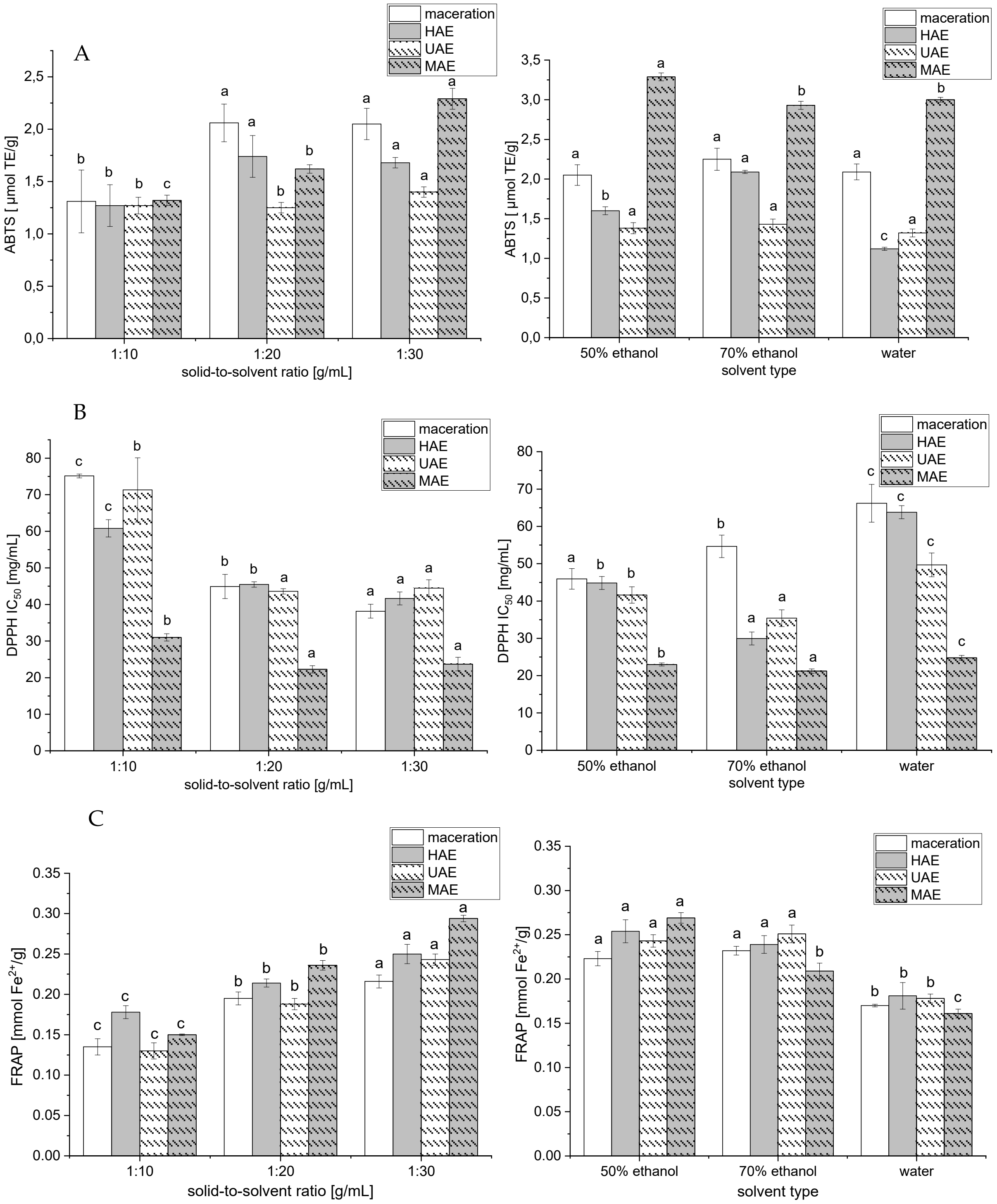
A
maceration
HAE
UAE
MAE
ABTS [µmol TE/g]
1:10
1:20
1:30
solid-to-solvent ratio [g/mL]
50% ethanol
70% ethanol
water
solvent type
B
DPPH IC50 [mg/mL]
C
FRAP [mmol Fe2+/g]

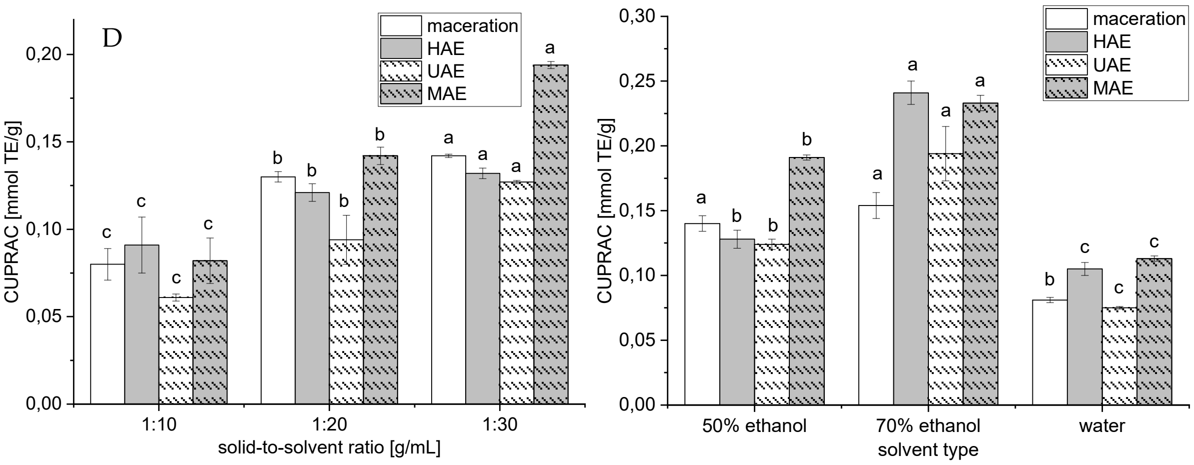

Figure S1. The influence of solid-to-solvent ratio and solvent type on (A) ABTS and (B) DPPH radical scavenging activity, (C) ferric reducing antioxidant power (FRAP), and (D) cupric ion-reducing antioxidant capacity (CUPRAC) of *Aloe vera* leaf waste extracts obtained in maceration, heat-, ultrasound-, and microwave-assisted extractions (HAE, UAE, and MAE, respectively).
