# Supplementary material for: From Aloe vera Leaf Waste to the Extracts with Biological Potential: Optimization of the Extractions, Physicochemical Characterization, and Biological Activities
Source: Plants (Basel). 2023 Jul 24;12(14):2744. doi: 10.3390/plants12142744 (PMC10386512; doi:10.3390/plants12142744)
Supplement: Supplementary file 1 [file plants-12-02744-s001.zip › Figure S2.pdf]

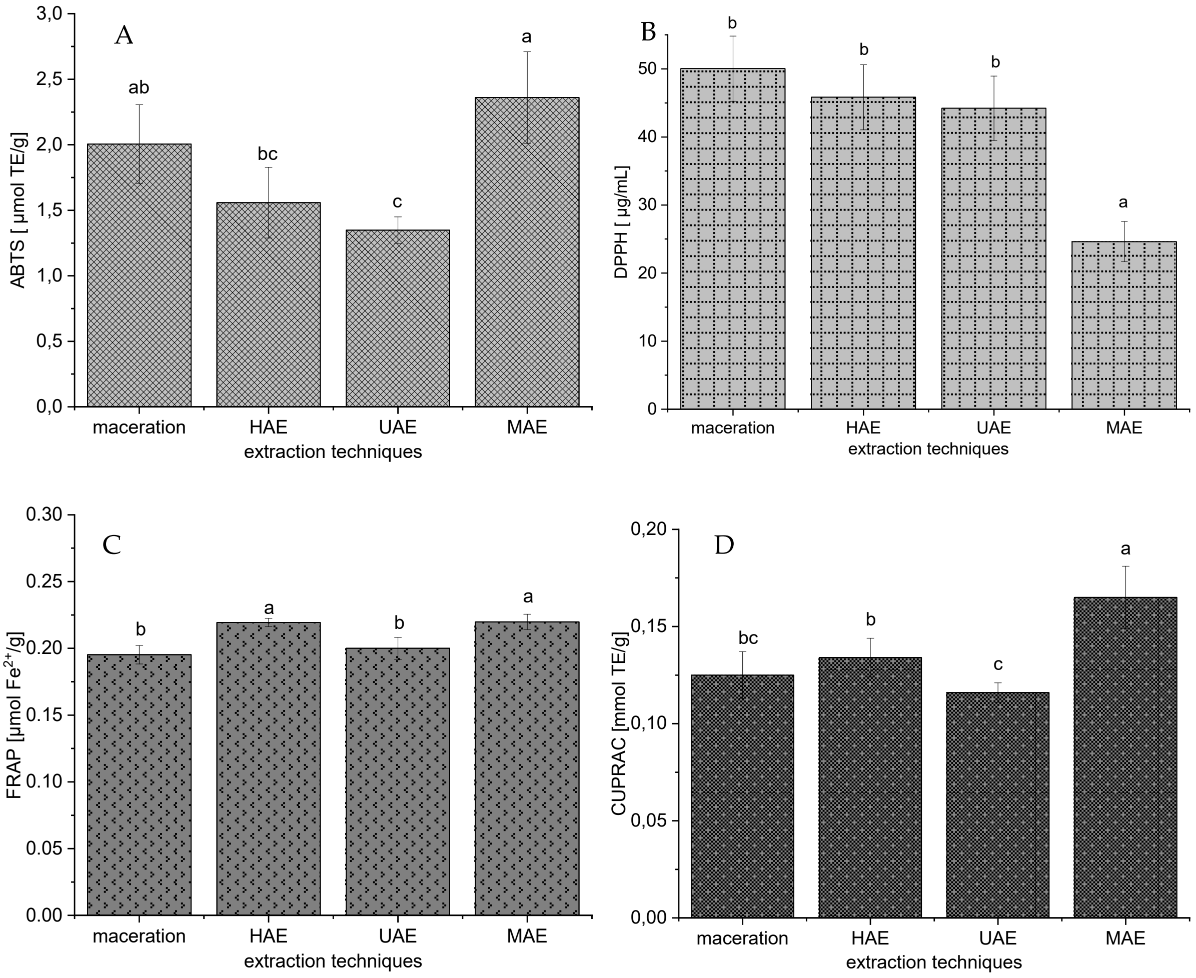

Figure S2. Effects of extraction techniques on (A) ABTS and (B) DPPH radical scavenging activity, (C) ferric reducing antioxidant power (FRAP), and (D) cupric ion-reducing antioxidant capacity (CUPRAC) of all prepared *Aloe vera* leaf waste extracts using maceration, heat-, ultrasound-, and microwave-assisted extractions (HAE, UAE, and MAE, respectively); different letters represent different population groups based on Duncan's *post hoc* test ($p<0.05$).
