# Supplementary material for: From Aloe vera Leaf Waste to the Extracts with Biological Potential: Optimization of the Extractions, Physicochemical Characterization, and Biological Activities
Source: Plants (Basel). 2023 Jul 24;12(14):2744. doi: 10.3390/plants12142744 (PMC10386512; doi:10.3390/plants12142744)
Supplement: Supplementary file 1 [file plants-12-02744-s001.zip › Figure S3.pdf]

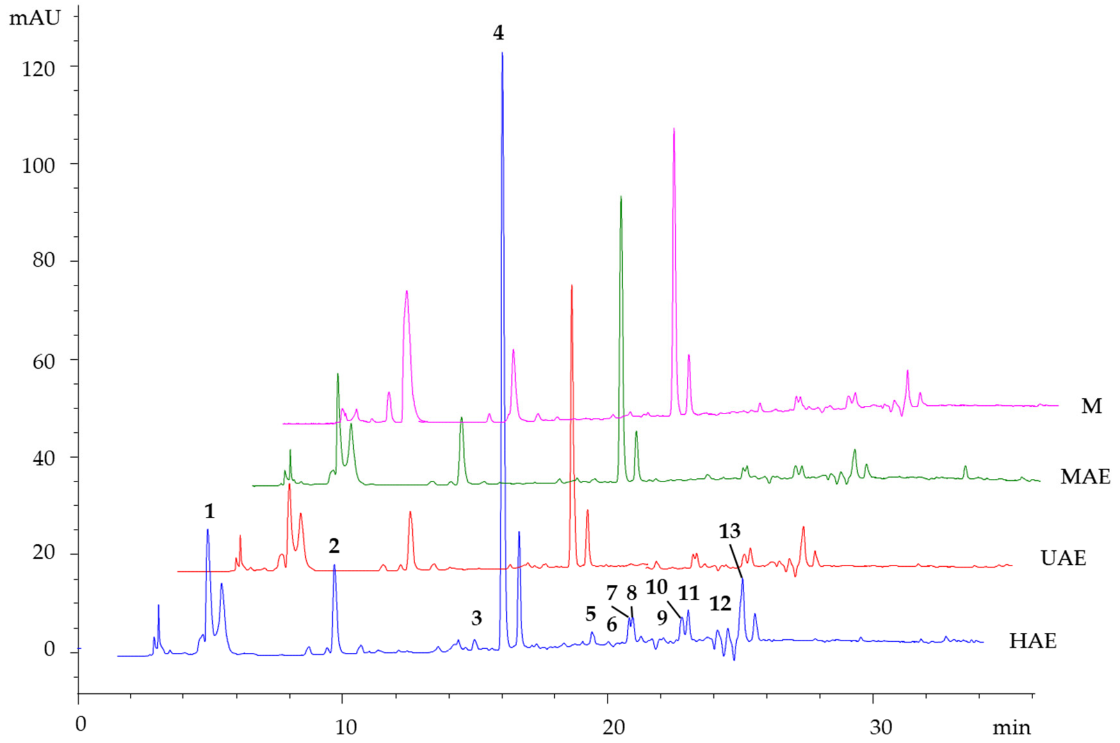

Figure 3a. Chromatograms of selected *Aloe vera* leaf waste extracts prepared using maceration, heat-, ultrasound- and microwave-assisted extractions (M, HAE, UAE, and MAE, respectively); **1**, unknown compound; **2**, hydroxymethyl aloin; **3**, malonylaloesin; **4**, aloin hexoside derivative; **5**, nataloin derivative; **6**, aloin hexoside derivative; **7**, aloin hexoside; **8**, aloe-emodin dihexoside; **9**, aloeresin, **10** aloin B; **11**, aloin A; **12**, malonylnataloin; **13**, aloe-emodin hexoside derivative.

Anthraquinones

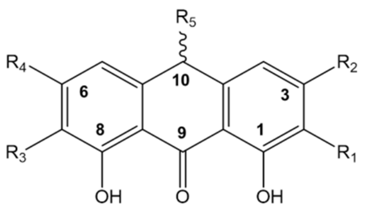

Compound	R_1	R_2	R_3	R_4	R_5
**2**	H	CH_2OH	H	CH_2OH	Glc
**10** and **11**	H	CH_2OH	H	H	Glc
**12**	OH	H	H	CH_3	Malonyl-Glc
**Aloe-emodin** (aglycone of **2**, **4**, **6-8**, **10**, **11**, **13**)	H	CH_2OH	H	H	=O

Chromones

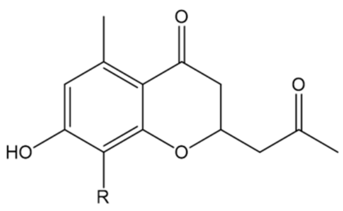

Compound	R
**3**	Malonyl-Glc
**9**	p-Coumaroyl-Glc

Figure 3b. Structure of the compounds identified in the selected *Aloe vera* leaf waste extracts using LC-MS analysis.
