# Supplementary material for: From Aloe vera Leaf Waste to the Extracts with Biological Potential: Optimization of the Extractions, Physicochemical Characterization, and Biological Activities
Source: Plants (Basel). 2023 Jul 24;12(14):2744. doi: 10.3390/plants12142744 (PMC10386512; doi:10.3390/plants12142744)
Supplement: Supplementary file 1 [file plants-12-02744-s001.zip › Table S1.pdf]

Table S1. Statistical analysis of optimization of maceration, heat-, ultrasound-, and microwave-assisted extractions (HAE, UAE, and MAE, respectively) from *Aloe vera* leaf waste using 2^3 factorial design.

	Effect	Std. Err.	Effect Estimates	Coeff.	Std. Err. Coeff.	*p*
**Maceration**						
TPC* [mg GAE/g]						
Constant				6.407	0.068	0.000
Main factors						
Solid-to-solvent ratio [g/mL] (1)	2.251	0.136	16.555	1.125	0.068	0.000
Solvent type (2)	-2.419	0.136	-17.794	-1.210	0.068	0.000
Time [min] (3)	1.849	0.136	13.601	0.925	0.068	0.000
Interaction of two factors						
1 by 2	-2.337	0.325	-6.221	-0.423	0.068	0.000
1 by 3	0.434	0.325	0.484	0.033	0.068	0.634
2 by 3	0.784	0.325	-0.619	-0.042	0.068	0.544
**HAE**						
TPC [mg GAE/g]						
Constant				9.314	0.055	0.000
Main factors						
Solid-to-solvent ratio [g/mL] (1)	1.031	0.109	9.431	0.515	0.055	0.000
Solvent type (2)	1.073	0.109	9.812	0.536	0.055	0.000
Time [min] (3)	0.096	0.109	0.877	0.048	0.055	0.393
Interaction of two factors						
1 by 2	-0.231	0.109	-2.112	-0.115	0.055	0.049
1 by 3	-0.061	0.109	-0.556	-0.030	0.055	0.585
2 by 3	0.171	0.109	1.563	0.085	0.055	0.136
**UAE**						
TPC [mg GAE/g]						
Constant				7.436	0.053	0.000
Main factors						
Solid-to-solvent ratio [g/mL] (1)	1.585	0.106	14.881	0.792	0.053	0.000
Solvent type (2)	1.618	0.106	15.194	0.809	0.053	0.000
Time [min] (3)	1.452	0.106	13.629	0.726	0.053	0.000
Interaction of two factors						
1 by 2	-0.628	0.106	-5.899	-0.314	0.053	0.000
1 by 3	-0.642	0.106	-6.024	-0.321	0.053	0.634
2 by 3	0.398	0.106	3.740	0.199	0.053	0.544
**MAE**						

TPC [mg GAE/g]						
Constant				7.687	0.068	0.000
Main factors						
Solid-to-solvent ratio [g/mL] (1)	1.410	0.135	10.417	0.705	0.068	0.000
Solvent type (2)	0.342	0.135	2.524	0.171	0.068	0.022
Time [min] (3)	0.890	0.135	6.575	0.445	0.068	0.000
Interaction of two factors						
1 by 2	0.105	0.135	0.776	0.052	0.068	0.448
1 by 3	0.057	0.135	0.419	0.028	0.068	0.681
2 by 3	-0.108	0.135	-0.800	-0.054	0.068	0.434

*TPC, total polyphenol content; GAE, gallic acid equivalent.
