# Supplementary material for: From Aloe vera Leaf Waste to the Extracts with Biological Potential: Optimization of the Extractions, Physicochemical Characterization, and Biological Activities
Source: Plants (Basel). 2023 Jul 24;12(14):2744. doi: 10.3390/plants12142744 (PMC10386512; doi:10.3390/plants12142744)
Supplement: Supplementary file 1 [file plants-12-02744-s001.zip › Table S2.pdf]

Table S2. The influence of extraction time on ABTS and DPPH radical scavenging activity, ferric reducing antioxidant power (FRAP), and cupric ion-reducing antioxidant capacity (CUPRAC) of *Aloe vera* leaf waste extracts obtained in maceration, heat-, ultrasound-, and microwave-assisted extractions (HAE, UAE, and MAE, respectively).

Extraction techniques	Time [min]	antioxidant capacity			
		ABTS [μmol TE/g]	DPPH IC_{50} [mg/mL]	FRAP [mmol Fe^{2+}/g]	CUPRAC [mmol TE/g]
Maceration	30	1.80±0.13[a*]	43.86±2.81[a]	0.200±0.006[b]	0.126±0.003[b]
	45	2.05±0.17[a]	41.08±4.13[a]	0.216±0.008[a]	0.140±0.004[a]
	60	2.01±0.18[a]	40.38±1.89[a]	0.210±0.005[ab]	0.128±0.002[b]
HAE	15	1.36±0.04[b]	42.68±1.32[a]	0.212±0.010[b]	0.125±0.008[a]
	30	1.52±0.08[a]	41.66±2.76[a]	0.250±0.012[a]	0.132±0.003[a]
	45	1.64±0.11[a]	41.57±1.25[a]	0.241±0.008[a]	0.133±0.010[a]
UAE	5	1.35±0.17[a]	42.56±2.44[b]	0.157±0.001[b]	0.112±0.003[b]
	15	1.40±0.20[a]	35.91±3.04[a]	0.243±0.007[a]	0.127±0.001[a]
	30	1.35±0.22[a]	33.38±1.10[a]	0.149±0.002[c]	0.125±0.007[a]
MAE	1	2.19±0.07[a]	29.38±1.60[b]	0.269±0.006[b]	0.173±0.008[b]
	2	2.30±0.07[a]	24.78±0.26[a]	0.294±0.004[a]	0.192±0.003[a]
	3	2.22±0.06[a]	24.87±1.06[a]	0.206±0.007[c]	0.164±0.005[b]

* Values with the same letter in each column showed no statistically significant difference (p>0.05; n=3; analysis of variance, Duncan's *post-hoc* test); TE, Trolox equivalents; IC_{50}, the concentration of extract required to neutralize 50% of DPPH radicals.
