# Supplementary material for: From Aloe vera Leaf Waste to the Extracts with Biological Potential: Optimization of the Extractions, Physicochemical Characterization, and Biological Activities
Source: Plants (Basel). 2023 Jul 24;12(14):2744. doi: 10.3390/plants12142744 (PMC10386512; doi:10.3390/plants12142744)
Supplement: Supplementary file 1 [file plants-12-02744-s001.zip › Table S3.pdf]

Table S3. The antimicrobial activity of selected *Aloe vera* leaf waste extracts prepared using maceration, heat-, ultrasound- and microwave-assisted extractions (HAE, UAE, and MAE, respectively) determined using disk diffusion method.

Microorganism	maceration	HAE	UAE	MAE	antibiotics/fluconazole
zone of inhibition [mm]					
Staphylococcus aureus	12±0.5[a]	11±0.4[a]	12±0.5[a]	11±0.7[a]	S
Enterococcus faecalis	3±0.3[b]	7±0.7[a]	6±0.4[a]	3±0.2[b]	S
Escherichia coli	R	R	R	R	R/I/S
Pseudomonas aeruginosa	R	R	R	R	S/I
Klebsiella spp.	R	R	R	R	S/I
Proteus spp.	R	R	R	R	S/I
Candida albicans	R	R	R	R	S

Values with the same letter in each row showed no statistically significant difference ($p>0.05$; $n=3$; analysis of variance, Duncan's *post-hoc* test); S, sensitive; I, intermediate sensitive; R, resistant.
